# Targeting Plasma Membrane Ca^2+^-ATPases in Cancer: Current Insights and Future Perspectives

**DOI:** 10.3390/cancers18152450

**Published:** 2026-07-30

**Authors:** Malwina Lisek, Julia Tomczak, Natalia Bochenska, Julia Duraj, Tomasz Boczek

**Affiliations:** Department of Molecular Neurochemistry, Medical University of Lodz, 90-419 Łódź, Poland; malwina.lisek@umed.lodz.pl (M.L.); julia.tomczak1@student.umed.lodz.pl (J.T.); natalia.bochenska@umed.lodz.pl (N.B.); julia.duraj@student.umed.lodz.pl (J.D.)

**Keywords:** plasma membrane Ca^2+^-ATPase, calcium signaling, cancer, calcium microdomains, tumor progression

## Abstract

Calcium signaling is a fundamental regulator of cell physiology, controlling proliferation, differentiation, migration, metabolism, gene expression, and cell death. In cancer, these signaling pathways are extensively remodeled to generate spatially and temporally restricted Ca^2+^ signals that support malignant progression while avoiding calcium-induced cytotoxicity. PMCAs traditionally regarded as high-affinity calcium extrusion pumps, have recently emerged as multifunctional regulators of compartmentalized calcium signaling. In addition to maintaining low cytosolic Ca^2+^ concentrations, PMCA isoforms organize specialized signaling microdomains by interacting with receptors, ion channels, scaffold proteins, and downstream signaling molecules, thereby selectively modulating calcium-dependent pathways involved in tumor growth and metastasis. Accumulating evidence demonstrates that PMCA isoforms exert distinct, context-dependent functions in cancer. PMCA1 primarily contributes to basal calcium homeostasis but has also been implicated in tumor progression, angiogenesis, and regulation of the tumor immune microenvironment. PMCA2 promotes survival and oncogenic signaling in HER2-positive breast cancer through stabilization of receptor signaling complexes. PMCA3 has been linked mainly to endocrine tumors and selected malignancies, although mechanistic evidence remains limited. PMCA4 exhibits the greatest functional diversity, acting either as a tumor suppressor or a promoter depending on the cancer type by regulating localized calcium signaling, cell migration, invasion, differentiation, and interactions with oncogenic signaling networks. This review summarizes current advances in the structural biology, regulation, and signaling functions of PMCA isoforms, with particular emphasis on their emerging roles in cancer biology. We also discuss the potential of PMCAs as prognostic biomarkers and therapeutic targets, highlighting the importance of isoform-specific strategies for targeting calcium signaling in cancer.

## Simple Summary

Calcium signaling is increasingly recognized as a key regulator of cancer development, and plasma membrane Ca^2+^-ATPases (PMCAs) have emerged as critical modulators of this process. Beyond maintaining intracellular Ca^2+^ homeostasis, PMCA isoforms organize localized calcium microdomains and assemble signaling complexes that regulate proliferation, migration, invasion, apoptosis, and therapy resistance. Recent studies demonstrate that PMCA isoforms exhibit distinct, context-dependent functions in cancer, acting either as tumor promoters or tumor suppressors depending on the malignancy and molecular environment. In particular, PMCA2 and PMCA4 regulate oncogenic signaling through compartmentalized Ca^2+^ signaling, while PMCA1 and PMCA3 display more specialized roles in selected cancers. This review summarizes current knowledge on the structure, regulation, and signaling functions of PMCAs, highlights their isoform-specific contributions to tumor progression, and discusses their potential as prognostic biomarkers and therapeutic targets in precision oncology.

## 1. Introduction

Calcium is a ubiquitous intracellular second messenger that regulates virtually every aspect of cell physiology, including proliferation, differentiation, metabolism, migration, gene expression, and cell death. The specificity of calcium signaling depends not only on changes in cytosolic Ca^2+^ concentration but also on the spatial and temporal organization of calcium signals into highly localized microdomains. These precisely coordinated calcium dynamics are generated through the integrated activity of plasma membrane channels, intracellular calcium release channels, ATP-driven pumps, exchangers, and calcium-binding proteins, collectively forming a complex signaling network that maintains cellular homeostasis.

The importance of calcium homeostasis becomes particularly evident in cancer, where dysregulated calcium signaling has emerged as a key hallmark of malignant transformation. Virtually every stage of tumor development is influenced by alterations in calcium signaling pathways. Rather than simply increasing intracellular Ca^2+^ levels, cancer cells remodel the entire calcium signaling toolkit to generate localized, stimulus-specific calcium signals that selectively activate pro-tumorigenic pathways while avoiding calcium overload and cell death. Advances in molecular biology, live-cell imaging, and genetically encoded calcium indicators have revealed that calcium signaling functions as a highly compartmentalized network, in which local calcium nanodomains generated at the plasma membrane, endoplasmic reticulum, mitochondria-associated membranes, focal adhesions, invadopodia, primary cilia, and lipid rafts activate specific downstream effectors without substantially altering global cytosolic calcium concentrations. This spatial organization enables cancer cells to independently regulate proliferation, migration, angiogenesis, metabolic adaptation, and communication with the tumor microenvironment [1,2].

Among the molecular components responsible for maintaining calcium homeostasis, PMCAs have emerged as critical regulators of both global and compartmentalized calcium signaling. As high-affinity calcium extrusion pumps, PMCAs maintain low resting cytosolic Ca^2+^ concentrations while shaping the amplitude, duration, and spatial characteristics of intracellular calcium signals. Together with sarco/endoplasmic reticulum Ca^2+^-ATPases (SERCAs), Na^+^/Ca^2+^ exchangers (NCXs), mitochondrial calcium transporters, and calcium-buffering proteins, PMCAs ensure precise control of intracellular calcium homeostasis [3,4]. In electrically excitable cells, particularly cardiomyocytes, NCX constitutes the major high-capacity Ca^2+^ extrusion pathway. Although NCX exhibits a lower affinity for Ca^2+^ than PMCA, its considerably higher transport capacity enables rapid removal of the large cytosolic Ca^2+^ loads generated during repetitive action potentials and excitation–contraction coupling [5]. However, increasing evidence demonstrates that many cancers selectively alter the expression and activity of these transport systems to sustain calcium signals that favor proliferation, migration, invasion, and resistance to apoptosis.

Importantly, the role of PMCAs extends far beyond calcium extrusion. Emerging evidence indicates that PMCA isoforms function as signaling hubs that assemble multiprotein complexes at specialized plasma membrane domains, thereby organizing compartmentalized calcium microdomains and coordinating oncogenic signaling pathways. Through interactions with membrane receptors, ion channels, cytoskeletal proteins, and intracellular signaling molecules, PMCAs regulate localized calcium signaling that controls numerous processes associated with tumor progression [6,7]. These functions are highly isoform- and context-dependent, with individual PMCA isoforms exerting distinct, and in some cases opposing, effects depending on the tumor type, cellular localization, and molecular environment.

The recognition of PMCAs as both calcium transporters and signaling organizers has fundamentally changed our understanding of their role in cancer biology. Rather than acting solely as homeostatic pumps, PMCAs are increasingly viewed as dynamic regulators of malignant cell behavior and important determinants of tumor progression. Consequently, PMCA isoforms have emerged as promising biomarkers of cancer prognosis and attractive therapeutic targets. A better understanding of their canonical transport function together with their non-canonical signaling activities will be essential for elucidating the contribution of calcium signaling to tumor biology and for developing novel calcium-based therapeutic strategies.

Despite growing evidence implicating PMCA isoforms in cancer, our understanding of their roles remains fragmented. Most studies have focused either on individual PMCA isoforms or on specific tumor types, with relatively little effort devoted to integrating these findings into a unified mechanistic framework. Furthermore, the traditional view of PMCAs as mere Ca^2+^ extrusion pumps has increasingly been challenged by evidence demonstrating that they function as organizers of compartmentalized calcium signaling through interactions with receptors, ion channels, scaffold proteins, and signaling enzymes. These non-canonical functions appear to underlie many of the context-dependent effects of PMCAs on tumor initiation, progression, metastasis, and therapeutic response. This review addresses these knowledge gaps by providing a comprehensive synthesis of isoform-specific functions of PMCA1–PMCA4 across human cancers, critically distinguishing correlative observations from mechanistic evidence, comparing canonical and non-canonical modes of PMCA action, and discussing how these emerging concepts may guide the development of novel therapeutic strategies targeting PMCA-dependent signaling networks.

## 2. Overall Structural Organization of PMCAs

PMCAs belong to the P-type ATPase superfamily, a large group of ion transporters that utilize the energy derived from ATP hydrolysis to drive the active transport of ions across biological membranes. Other members of this family include SERCAs, Na^+^/K^+^-ATPases, and H^+^/K^+^-ATPases. Like all P-type ATPases, PMCAs form a transient phosphorylated intermediate during their catalytic cycle and undergo extensive conformational changes that couple ATP hydrolysis to ion translocation. However, PMCAs possess several structural adaptations that distinguish them from other members of the family, most notably an extended C-terminal regulatory domain that mediates autoinhibition and enables sophisticated signal-dependent regulation of pump activity [8,9].

In mammals, four genes encode PMCA isoforms: ATP2B1, ATP2B2, ATP2B3, and ATP2B4, which give rise to PMCA1, PMCA2, PMCA3, and PMCA4, respectively. Although these isoforms share a highly conserved overall architecture and exhibit approximately 75–85% amino acid sequence identity, they differ markedly in their tissue distribution, regulatory characteristics, and physiological functions. PMCA1 is ubiquitously expressed and is generally regarded as the housekeeping isoform responsible for maintaining basal intracellular Ca^2+^ homeostasis in most cell types. In contrast, PMCA2 and PMCA3 are enriched in excitable tissues, including neurons and sensory cells, where rapid and efficient calcium clearance is required. PMCA4 is also widely expressed but has attracted considerable attention because of its ability to function as a signaling scaffold, organizing calcium-dependent signaling complexes through interactions with numerous regulatory and signaling proteins [10,11].

**Figure 1:**
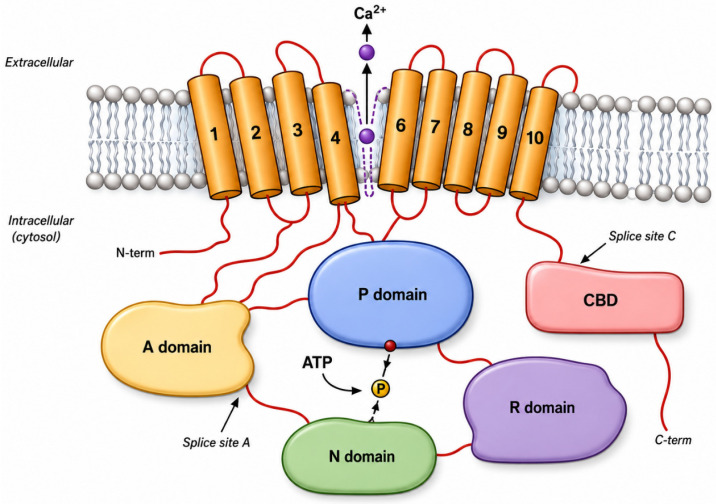
Structural organization of PMCA. The protein consists of ten transmembrane helices (TM1–TM10) forming the membrane domain, which contains the Ca^2+^ translocation pathway. The large cytoplasmic catalytic core is composed of three conserved domains: the actuator (A) domain, which coordinates conformational changes during the transport cycle; the phosphorylation (P) domain, containing the conserved aspartate residue that undergoes transient phosphorylation; and the nucleotide-binding (N) domain, which binds ATP and catalyzes phosphoryl transfer. The extended C-terminal regulatory region includes the calmodulin-binding domain (CBD) and the regulatory (R) domain, which mediates autoinhibition and integrates regulatory signals. Alternative splicing at sites A and C generates numerous PMCA variants with distinct regulatory properties and tissue-specific expression patterns.

The overall structure of PMCA consists of a single polypeptide chain comprising approximately 1200–1350 amino acids with a molecular mass of 125–140 kDa (Figure 1). Sequence homology with other P-type ATPases predicts the presence of ten transmembrane α-helices connected by intracellular and extracellular loops, a corresponding set of membrane-proximal stalk regions on the cytoplasmic side, and three major cytoplasmic domains that together form the catalytic core of the enzyme. These domains include the nucleotide-binding (N) domain, which binds ATP; the phosphorylation (P) domain, which contains a highly conserved aspartate residue that becomes transiently phosphorylated during the transport cycle; and the actuator (A) domain, which coordinates the conformational rearrangements required for ion transport and enzyme dephosphorylation. The coordinated movements of these domains enable the conversion of chemical energy derived from ATP hydrolysis into the mechanical work necessary for Ca^2+^ extrusion.

The transmembrane region forms the ion translocation pathway and contains the amino acid residues responsible for calcium coordination. Unlike SERCA pumps, which transport two Ca^2+^ ions per catalytic cycle, PMCAs bind and extrude a single Ca^2+^ ion during each transport cycle. This lower transport stoichiometry is accompanied by a substantially higher affinity for Ca^2+^, allowing PMCAs to function effectively under resting or moderately elevated intracellular calcium concentrations and thereby maintain precise control of cytosolic Ca^2+^ levels. Structural modeling studies have revealed additional features that distinguish PMCAs from other P-type ATPases. A nearly continuous positively charged ring has been identified within the membrane-proximal stalk region of PMCA, a structural element absent in SERCA pumps. This positively charged region is consistent with the “positive-inside rule” characteristic of many plasma membrane proteins and may contribute to proper membrane orientation, stabilization of protein structure, and interactions with negatively charged phospholipid head groups. Such features likely reflect evolutionary adaptations that optimize PMCA function within the unique lipid environment of the plasma membrane.

The transmembrane domain forms the membrane-embedded portion of the protein and consists of ten α-helical segments designated TM1 through TM10. These helices create the pathway through which calcium ions are transported across the plasma membrane. Structural and mutagenesis studies have demonstrated that the primary calcium-binding site is formed by amino acid residues located within transmembrane segments TM4, TM5, TM6, and TM8. Several highly conserved acidic residues, including aspartate and glutamate residues, coordinate calcium binding and stabilize the ion during transport. The geometry of these residues generates a high-affinity calcium-binding environment capable of recognizing cytosolic calcium concentrations within the nanomolar to low micromolar range [12,13].

As integral membrane proteins, PMCAs are highly dependent on their lipid surroundings for optimal activity [14,15]. Experimental reconstitution studies demonstrated that incorporation of purified PMCA into neutral phospholipid bilayers significantly enhances enzyme turnover, indicating that membrane lipids directly influence pump function. However, maximal catalytic activity requires the presence of acidic phospholipids, including phosphatidylserine and phosphatidylinositol derivatives, which further stimulate Ca^2+^ transport efficiency and stabilize functionally relevant conformational states of the enzyme. These observations suggest that specific lipid–protein interactions are essential for maintaining PMCA activity and may contribute to the localization of PMCA molecules within specialized plasma membrane microdomains.

## 3. Cytoplasmic Catalytic Core

The catalytic core of PMCA is located primarily within the large intracellular loop connecting transmembrane segments TM4 and TM5. Like all P-type ATPases, this region contains three highly conserved functional domains forming the molecular machinery responsible for ATP-dependent calcium transport. The phosphorylation domain contains a highly conserved aspartate residue located within the characteristic DKTG motif. During the transport cycle, ATP transfers a phosphate group to this aspartate residue, generating a transient phosphorylated intermediate that drives the conformational changes required for calcium translocation. The nucleotide-binding domain binds ATP and positions it appropriately for phosphate transfer to the catalytic aspartate. Structural analyses have revealed that ATP binding induces substantial conformational rearrangements that bring the nucleotide-binding and phosphorylation domains into close proximity, facilitating efficient catalysis. The actuator domain functions as a mechanical coupling element linking.

ATP hydrolysis to movements within the transmembrane region. During the catalytic cycle, the actuator domain participates in dephosphorylation of the phosphorylated intermediate and coordinates conformational transitions between the major transport states. Through these coordinated movements, chemical energy derived from ATP hydrolysis is converted into directed ion transport across the plasma membrane.

**Figure 2:**
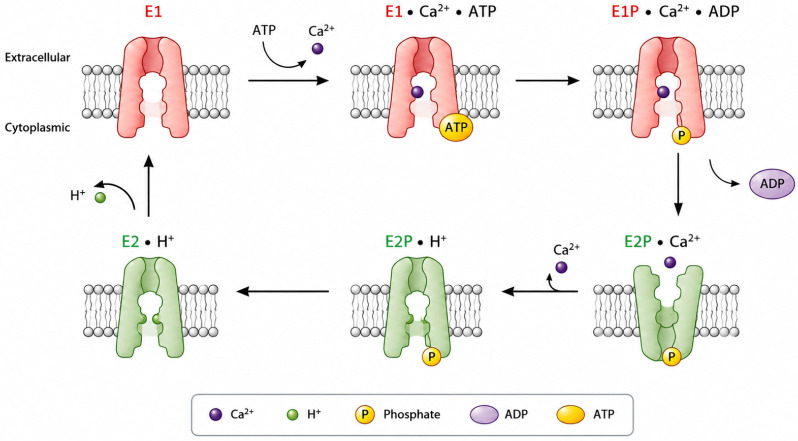
Post–Albers transport cycle of PMCA. PMCA transports a single Ca^2+^ ion across the plasma membrane during each catalytic cycle through alternating E1 and E2 conformational states. In the E1 conformation, the pump exhibits high affinity for cytosolic Ca^2+^ and binds one Ca^2+^ ion together with ATP. ATP hydrolysis results in phosphorylation of the conserved aspartate residue within the P domain, generating the E1P intermediate and occluding the bound Ca^2+^ ion. Release of ADP promotes the transition to the E2P conformation, in which the Ca^2+^-binding site is exposed to the extracellular side and displays low affinity for Ca^2+^, allowing ion release. Subsequently, one extracellular proton binds to the transporter, stabilizing the E2P state. Dephosphorylation produces the E2·H^+^ intermediate, followed by proton release into the cytoplasm and restoration of the high-affinity E1 conformation, thereby completing the transport cycle.

The transport mechanism of PMCA follows the classical Post–Albers cycle characteristic of P-type ATPases (Figure 2). In the E1 conformation, the calcium-binding site is accessible from the cytoplasmic side of the membrane and exhibits high affinity for calcium ions. Binding of calcium promotes ATP-dependent phosphorylation of the catalytic aspartate residue, leading to conformational changes that occlude the bound ion within the protein. Subsequent transition to the E2 state decreases calcium affinity and exposes the binding site to the extracellular environment, allowing calcium release. Dephosphorylation then restores the original E1 conformation, completing the transport cycle [16,17].

**Figure 3:**
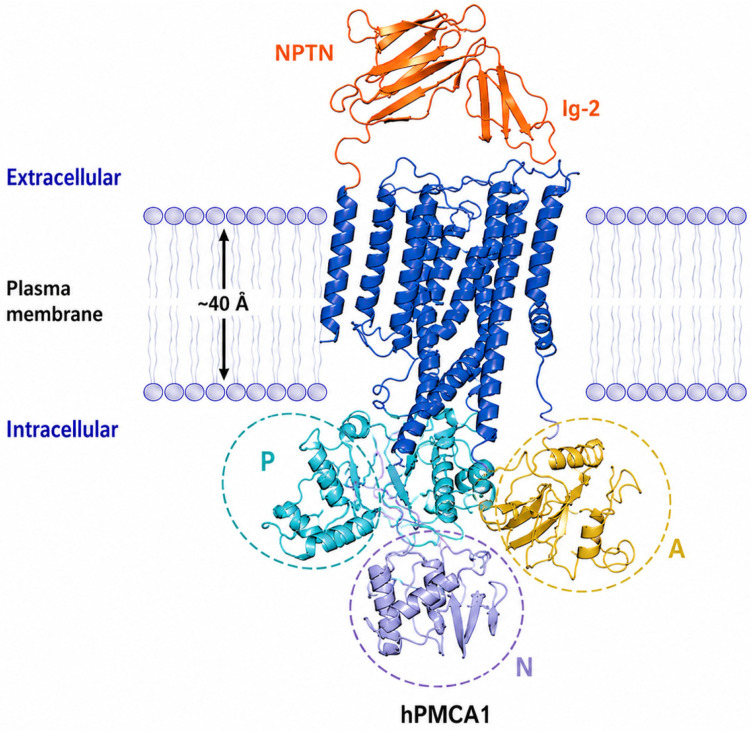
Overall architecture of human plasma membrane Ca^2+^-ATPase 1 (hPMCA1) in complex with neuroplastin (NPTN). The extracellular immunoglobulin-like domains of neuroplastin (NPTN), including the Ig-2 domain, interact with the transmembrane region of PMCA1 and are essential for pump maturation and activity. PMCA1 contains ten transmembrane α-helices that form the Ca^2+^ translocation pathway across the membrane. The cytoplasmic catalytic core is composed of the phosphorylation (P), actuator (A), and nucleotide-binding (N) domains, which coordinate ATP hydrolysis and the conformational changes underlying Ca^2+^ transport according to the Post–Albers transport cycle. The approximate thickness of the plasma membrane (~40 Å) is indicated.

Recent cryo-electron microscopy studies have provided unprecedented structural insight into this process by resolving mouse PMCA2 in eight distinct intermediates spanning the entire transport cycle. These structures confirmed the fundamental principles of the Post–Albers mechanism while revealing the molecular basis of the exceptionally rapid transport kinetics that distinguish PMCAs from other P-type ATPases. In particular, the study demonstrated that, in PMCA2, the plasma membrane phospholipid phosphatidylinositol 4,5-bisphosphate (PIP_2_) plays a critical role in stabilizing calcium-bound conformations and facilitating rapid ion release during the E1–E2 transition. Whether this mechanism is conserved across other PMCA isoforms remains to be established. This lipid-mediated regulation substantially accelerates conformational cycling and contributes to the transport rates observed for PMCA in living cells. Furthermore, the structures revealed how the auxiliary subunit neuroplastin associates with PMCA throughout the catalytic cycle, stabilizing the transporter and supporting efficient calcium extrusion (Figure 3). Together, these findings establish the first comprehensive structural model of the PMCA transport cycle and identify lipid–protein interactions as key determinants of the ultrafast calcium pumping activity that enables PMCAs to maintain the steep calcium gradient across the plasma membrane [18].

## 4. Alternative Splicing and Structural Diversity

The functional diversity of the PMCA family is substantially expanded through alternative RNA splicing at two major regulatory regions, known as splice sites A and C (see Figure 1 and Table 1). Combinatorial utilization of alternative exons at these loci generates more than twenty distinct PMCA variants, thereby increasing both structural and functional heterogeneity. Splicing at site A modifies the length of the first intracellular loop, whereas splicing at site C affects the composition of the C-terminal regulatory region. The resulting splice variants exhibit distinct developmental, tissue-specific, and cell-type-specific expression patterns, indicating that they have evolved to meet the unique calcium-handling requirements of different cellular environments.

Alternative splicing at site A introduces considerable structural variability by altering the size of the first cytoplasmic loop. In PMCA2, for example, the inclusion of the so-called w insert adds up to 45 amino acids to this region. Although high-resolution structural information for full-length PMCA isoforms remains limited, such differences are predicted to influence local protein architecture and potentially affect overall protein conformation. Consistent with this notion, splice variants containing the “w” insert are preferentially targeted to the apical membrane of polarized cells, whereas variants harboring the shorter “x” insert localize predominantly to lateral membrane domains [10,21].

Even greater structural diversity arises from alternative splicing at site C. Differential exon usage at this locus induces a reading-frame shift, generating splice variants that differ substantially in both the length and amino acid composition of their C-terminal tails. Consequently, the major “a” and “b” splice variants of each PMCA isoform possess distinct regulatory domains with different capacities for intramolecular interactions, particularly those involved in autoinhibition of the catalytic core. These structural differences also alter the ability of PMCA variants to interact with calmodulin (CaM), scaffolding proteins, signaling molecules, and other regulatory partners. As a result, alternative splicing contributes significantly to the functional specialization of PMCA isoforms, enabling precise regulation of calcium extrusion and signaling in a tissue- and cell-specific manner [8,10,21].

## 5. Functional Diversity of PMCA Isoforms

Although all PMCA isoforms function as high-affinity Ca^2+^ extrusion systems, they exhibit distinct functional properties that enable them to differentially regulate intracellular calcium signaling. These differences arise largely from variations in their regulatory domains, alternative splice variants, membrane localization, and kinetic responses to calcium and CaM. Consequently, individual PMCA isoforms are adapted to the specific calcium-handling requirements of different cell types and subcellular compartments.

Perhaps the most distinctive structural feature of PMCA is its extended cytoplasmic C-terminal regulatory domain. This region is absent or significantly less elaborate in many other P-type ATPases and provides the molecular basis for the sophisticated regulation that characterizes PMCA activity. Under resting conditions, the distal C-terminal tail interacts with the catalytic core of the enzyme, stabilizing an autoinhibited conformation associated with low transport activity. This autoinhibitory mechanism prevents unnecessary ATP consumption and allows the pump to remain poised for rapid activation when intracellular calcium levels rise [23]. Biochemical and structural studies have demonstrated that the autoinhibitory region interacts with multiple domains within the catalytic core, thereby reducing ATPase activity and calcium transport efficiency. Embedded within the C-terminal tail is the calmodulin-binding domain, which represents the primary mechanism by which PMCA activity is regulated in response to changes in intracellular calcium concentration. CaM is a ubiquitous calcium-binding protein that serves as one of the major intracellular calcium sensors. When intracellular calcium levels increase, calcium ions bind to CaM, inducing conformational changes that enable it to associate with the PMCA C-terminus. This interaction displaces the autoinhibitory region from the catalytic core, resulting in a dramatic increase in pump activity [24,25,26].

CaM is the principal physiological activator of PMCA and enhances pump function primarily by dramatically increasing its affinity for Ca^2+^. Upon calmodulin binding, the apparent Km for Ca^2+^ decreases from approximately 10–20 μM to below 0.5 μM, allowing the pump to operate efficiently at much lower intracellular calcium concentrations [27]. In biochemical assays using purified PMCA preparations, CaM typically increases ATPase activity by approximately four- to six-fold under saturating Ca^2+^ conditions. Depending on the isoform and splice variant, CaM binding can stimulate PMCA activity by more than an order of magnitude (Table 2). For example, PMCA4a exhibits significantly faster activation by Ca^2+^-CaM than PMCA4b, allowing it to respond more efficiently to rapid calcium transients. In contrast, PMCA4b activates and deactivates more slowly, making it better suited for controlling prolonged or tonic elevations of intracellular Ca^2+^. This elegant feedback mechanism allows rising intracellular calcium concentrations to activate the transporters responsible for restoring calcium homeostasis, thereby ensuring efficient termination of calcium signals. Differences in activation and inactivation kinetics are particularly important for the decoding of repetitive calcium signals. Because CaM dissociates from different PMCA isoforms at different rates, some pumps retain a form of “molecular memory” of previous activation [28,29].

PMCA2 represents a notable exception to this general regulatory mechanism. Unlike other PMCA isoforms, PMCA2 displays substantial basal activity even in the absence of CaM and undergoes only modest additional activation upon CaM binding. Although the structural basis for this unusual behavior remains unresolved, it is likely related to the specialized physiological functions of PMCA2 in cells that require exceptionally efficient calcium clearance, such as neurons and sensory hair cells [33,34].

In addition to CaM, PMCA activity is strongly influenced by acidic phospholipids. Binding of these lipids enhances PMCA function by increasing its apparent affinity for Ca^2+^, often reducing the Km to values comparable to those achieved through CaM activation [35]. Although the physiological significance of this regulation remains incompletely understood, the concentration of acidic phospholipids within the plasma membrane is thought to be sufficient to maintain a substantial degree of basal PMCA activation under normal cellular conditions. Several additional mechanisms can further modulate PMCA activity, although their physiological importance appears to be more limited. These include pump dimerization, which involves interactions within the calmodulin-binding region, as well as phosphorylation by protein kinase A (PKA) and protein kinase C (PKC). In contrast, proteolytic regulation may have greater pathological relevance. Specifically, cleavage of the C-terminal regulatory region by the calcium-dependent protease calpain removes the autoinhibitory calmodulin-binding domain, resulting in constitutive pump activation. This irreversible modification eliminates normal regulatory control and can promote continuous calcium extrusion, potentially disrupting cellular calcium homeostasis under pathological conditions [3,7,36].

## 6. PMCA Physiology and Pathophysiology

### 6.1. PMCA in the Nervous System

The nervous system exhibits the highest diversity and abundance of plasma membrane PMCA isoforms among mammalian tissues, reflecting the extraordinary requirement for precise temporal and spatial regulation of intracellular Ca^2+^ signaling. Neurons utilize transient elevations of cytosolic Ca^2+^ to regulate neurotransmitter release, dendritic growth, synaptic plasticity, gene expression, and activity-dependent survival. Because prolonged Ca^2+^ elevation is neurotoxic, these signaling events must be rapidly terminated while preserving the fidelity of subsequent neuronal responses. Owing to its exceptionally high affinity for Ca^2+^, PMCA represents one of the principal Ca^2+^ extrusion mechanisms responsible for restoring resting intracellular Ca^2+^ concentrations following physiological neuronal activity and for shaping local Ca^2+^ microdomains at the plasma membrane.

PMCA expression is tightly regulated throughout nervous system development. Although all four PMCA isoforms are ultimately expressed in the mature brain, their temporal and spatial expression patterns differ considerably, suggesting specialized developmental functions [37,38,39,40,41]. PMCA1 is expressed early during neurogenesis and provides basal Ca^2+^ homeostasis in differentiating neurons. During postnatal maturation, PMCA1a gradually replaces PMCA1b, coinciding with synaptic maturation. In contrast, PMCA2 expression increases later during development, particularly within the cerebellum, whereas PMCA4 expression remains relatively low during embryonic development and becomes more abundant in the adult brain. These developmental changes parallel periods of intense synaptogenesis and are accompanied by increased expression of calmodulin, phosphatidylserine, and protein kinase C, all of which positively regulate PMCA activity [42,43,44]. Functional studies further demonstrate that PMCA activity is required for normal neuronal differentiation. Suppression of PMCA2 or PMCA3 in differentiating PC12 cells delays neurite extension and reduces neuronal survival, whereas altered expression of individual isoforms induces compensatory changes in other PMCA family members [45,46]. These findings indicate that PMCA isoforms are not merely housekeeping Ca^2+^ pumps but actively participate in the regulation of neuronal morphogenesis and maturation.

Although PMCA isoforms partially overlap in their distribution, they are not functionally redundant. Purkinje neurons provide perhaps the best example of PMCA specialization. These cells express multiple PMCA isoforms simultaneously, with PMCA2 being highly enriched along the plasma membrane of the soma and throughout the extensive dendritic arbor, whereas PMCA1 and PMCA3 exhibit more restricted compartmental localization [37,40]. This remarkable diversity likely reflects the exceptionally complex Ca^2+^ dynamics required for cerebellar signal integration and motor coordination. Purkinje cells also contain high levels of other Ca^2+^-handling proteins, including SERCA2, SPCA1, calbindin, and parvalbumin, highlighting the importance of coordinated Ca^2+^ buffering and extrusion mechanisms within these neurons [47,48,49].

One of the most important physiological functions of PMCA in neurons is the termination of Ca^2+^-dependent signaling. PMCA isoforms are strategically localized within dendritic spines and the postsynaptic density, positioning them to efficiently remove Ca^2+^ following synaptic activation. Their high expression in pyramidal neurons is thought to underlie the remarkable capacity of these cells for rapid Ca^2+^ clearance [50,51]. Importantly, PMCA activity is dynamically regulated by intracellular Ca^2+^ levels. During sustained neuronal stimulation, elevated Ca^2+^ concentrations reduce the efficiency of PMCA-mediated extrusion, leading to slower recovery of basal Ca^2+^ levels [32]. This decrease in transport activity may partially result from Ca^2+^-dependent proteolytic modification of PMCA, although additional reversible regulatory mechanisms likely contribute to the transient slowing of Ca^2+^ clearance observed during repetitive neuronal firing [32]. Together with Na^+^/Ca^2+^ exchangers, which are also enriched in dendrites and dendritic spines [52], PMCAs cooperate to shape the amplitude and duration of local postsynaptic Ca^2+^ signals.

PMCA-dependent regulation of Ca^2+^ is also particularly important during long-term potentiation, one of the principal cellular mechanisms underlying learning and memory. LTP requires precisely timed activation of NMDA receptors, CaMKII, PKA, MAPK, CREB, and other signaling molecules in response to transient postsynaptic Ca^2+^ elevations [53]. Delayed Ca^2+^ clearance prolongs activation of these pathways and alters the balance between kinase- and phosphatase-dependent signaling. In particular, excessive activation of CaN antagonizes CaMKII-mediated phosphorylation events, shifting synaptic responses toward long-term depression rather than potentiation. By terminating local Ca^2+^ signals with high temporal precision, PMCA preserves the appropriate activation window for downstream signaling molecules and contributes to the fidelity of activity-dependent synaptic remodeling.

The physiological importance of PMCA is underscored by its involvement in neuronal pathophysiology. Ageing is accompanied by a progressive decline in synaptic PMCA activity and reduced calmodulin levels, resulting in impaired Ca^2+^ extrusion, prolonged intracellular Ca^2+^ elevations, and increased susceptibility of neurons to excitotoxicity [54,55]. Similar alterations have been observed following cerebral ischemia [56], where reduced PMCA expression contributes to sustained Ca^2+^ overload and neuronal injury. PMCA dysfunction has also been implicated in major neurodegenerative disorders, including Alzheimer’s [57,58] and Huntington’s diseases [59]. In Alzheimer’s disease, amyloid-β directly inhibits PMCA activity and alters its Ca^2+^ sensitivity, whereas oxidative stress further compromises pump function through oxidative modification of the protein. Likewise, reduced PMCA2 expression has been reported in Huntington’s disease, linking impaired Ca^2+^ extrusion to neuronal Ca^2+^ dyshomeostasis and progressive neurodegeneration. Collectively, these findings demonstrate that disruption of PMCA-mediated Ca^2+^ transport represents a common mechanism contributing to neuronal dysfunction across diverse neurological disorders.

### 6.2. Sensory Hair Cells

Sensory hair cells of the cochlea and vestibular system represent one of the most specialized examples of PMCA-dependent Ca^2+^ homeostasis. During mechanotransduction, deflection of the stereociliary bundle opens mechanotransduction channels, allowing rapid Ca^2+^ entry into the stereocilia. Because stereocilia lack intracellular Ca^2+^ stores and the endolymph contains extremely low Ca^2+^ concentrations, efficient Ca^2+^ extrusion is essential to rapidly restore basal Ca^2+^ levels and preserve the fidelity of mechanotransduction. Under these conditions, PMCA serves as the principal Ca^2+^ extrusion mechanism [60,61,62].

Hair cells exhibit remarkable isoform-specific compartmentalization of PMCA [63,64]. PMCA1 is predominantly localized to the basolateral membrane, whereas the stereociliary bundle contains almost exclusively PMCA2, particularly the PMCA2a and PMCA2w/a splice variants. This complete segregation indicates that hair cells tailor Ca^2+^ extrusion to distinct cellular compartments, with PMCA2 specialized for regulating the unique Ca^2+^ microenvironment of stereocilia while PMCA1 maintains global cellular Ca^2+^ homeostasis.

PMCA2 possesses biochemical properties ideally suited for mechanotransduction. Unlike other PMCA isoforms, PMCA2 displays exceptionally high basal transport activity and only modest stimulation by calmodulin, enabling continuous Ca^2+^ extrusion even during repetitive sensory stimulation. The predominant stereociliary splice variant, PMCA2w/a, is expressed at an exceptionally high density, approximately 2000 pumps per μm^2^ of stereociliary membrane, providing sufficient transport capacity to rapidly remove Ca^2+^ entering through mechanotransduction channels [65]. Beyond restoring Ca^2+^ homeostasis, PMCA2 directly regulates the physiology of mechanotransduction. Local Ca^2+^ concentrations within stereocilia determine the adaptation kinetics and sensitivity of these channels, influence active hair-bundle movements, and contribute to maintaining the molecular architecture of the mechanotransduction apparatus. By rapidly extruding Ca^2+^ immediately, PMCA2 shapes highly localized Ca^2+^ microdomains that fine-tune sensory transduction while preventing cytotoxic Ca^2+^ accumulation [66,67].

The physiological importance of PMCA2 is further illustrated by genetic and functional studies [68,69]. Mouse models lacking functional PMCA2 exhibit elevated stereociliary Ca^2+^, impaired mechanotransduction, reduced sensitivity of mechanotransduction channels, progressive degeneration of the organ of Corti, and severe auditory and vestibular dysfunction [70]. Similar phenotypes have been described in humans carrying ATP2B2 mutations [71,72,73], demonstrating that disruption of PMCA2-mediated Ca^2+^ extrusion compromises not only hair-cell signaling but also long-term cell survival. These observations establish PMCA2 as the central regulator of Ca^2+^ homeostasis in sensory hair cells and one of the most specialized PMCA isoforms in mammalian physiology.

### 6.3. Mammary Glands

Early studies demonstrated that PMCA expression increases dramatically during lactation in parallel with milk production. Among all PMCA isoforms, PMCA2 becomes the predominant calcium pump in secretory mammary epithelial cells, whereas PMCA4 predominates during mammary gland development and declines after the onset of lactation. PMCA1 expression increases only modestly, indicating that the transition to active milk secretion is accompanied by a highly selective induction of PMCA2 rather than a general increase in plasma membrane Ca^2+^ pumps. Molecular analyses further identified a mammary gland-specific splice variant, PMCA2bw, generated by alternative splicing at site A. This splice variant is specifically targeted to the apical membrane of secretory epithelial cells, where it is ideally positioned to mediate Ca^2+^ secretion into milk [74,75].

The discovery of apically localized PMCA2 fundamentally changed the classical model of mammary calcium secretion. Traditionally, milk calcium was believed to reach the lumen almost exclusively through the Golgi-dependent secretory pathway, where calcium associates with casein-containing secretory vesicles. However, the marked induction of PMCA2 during lactation, together with its localization at the apical membrane, demonstrated that a substantial proportion of calcium is transported directly across the plasma membrane into milk. Current evidence indicates that approximately 60–70% of milk calcium is exported by PMCA2, whereas the remaining fraction is delivered through the Golgi apparatus via the secretory pathway Ca^2+^ pumps SPCA1 and SPCA2 [76,77]. Thus, calcium secretion into milk results from coordinated activity of both apical plasma membrane transport and intracellular secretory pathways.

The physiological importance of PMCA2 was unequivocally demonstrated using PMCA2-deficient mice [75]. Despite normal expression of major milk proteins, milk produced by knockout animals contained approximately 60% less calcium than that of wild-type littermates and was associated with reduced milk production. These findings established PMCA2 as the principal transporter responsible for calcium secretion into milk and revealed a previously unrecognized physiological role for PMCA—direct transepithelial transport of large amounts of Ca^2+^ rather than merely maintaining intracellular Ca^2+^ homeostasis.

PMCA2 also contributes to mammary gland remodeling following lactation. Upon weaning, PMCA2 expression declines rapidly, preceding the onset of epithelial apoptosis. Loss of PMCA2 disrupts intracellular Ca^2+^ homeostasis, causing intracellular Ca^2+^ accumulation and sensitizing mammary epithelial cells to apoptosis. This transient “calcium crisis” appears to represent one of the earliest signals initiating mammary gland involution. Conversely, sustained PMCA2 expression protects mammary epithelial cells from apoptosis and, in breast cancer cells, promotes survival by maintaining low intracellular Ca^2+^ concentrations [77,78].

Collectively, studies of the mammary gland have expanded the classical view of PMCA function. Rather than serving as a high-affinity Ca^2+^ extrusion pump that shapes intracellular signaling, PMCA2 functions as a specialized epithelial transporter responsible for large-scale Ca^2+^ secretion. The mammary gland therefore provides one of the clearest physiological examples of how isoform-specific expression, alternative splicing, polarized membrane targeting, and extracellular signaling converge to adapt PMCA function to the specialized demands of a particular tissue.

### 6.4. Polarized Epithelial Transport

Unlike most membrane transporters, PMCA isoforms exhibit highly specialized membrane targeting. PMCA1 and PMCA4 generally localize to the basolateral membrane, where they participate in transcellular Ca^2+^ transport and extrusion into the interstitial space. In contrast, specific PMCA2 splice variants are targeted to the apical membrane, allowing direct regulation of luminal Ca^2+^ concentrations in specialized epithelia such as mammary gland, inner ear sensory epithelia, and kidney tubules. This polarized distribution enables epithelial cells to maintain directional Ca^2+^ transport while preserving intracellular Ca^2+^ homeostasis [79,80].

A key breakthrough was the discovery that membrane targeting is dictated primarily by alternative splicing at splice site A. Among PMCA2 splice variants, only the PMCA2w isoforms, which contain a unique 45-amino-acid insertion within the first intracellular loop, undergo efficient apical targeting. In contrast, the x, y, and z variants remain predominantly basolateral regardless of their C-terminal splice configuration. Importantly, exchanging the C-terminal PDZ-binding sequence does not alter this localization, demonstrating that the apical targeting information resides within the w insert itself [79]. Subsequent studies further demonstrated that the “w” insert functions autonomously. Insertion of the PMCA2 w-sequence into PMCA4 redirected this normally basolateral pump to the apical membrane without affecting its transport kinetics, proving that the targeting sequence acts independently of the catalytic properties of the pump. Thus, membrane localization and calcium transport activity can be regulated separately, allowing epithelial cells to position identical transport machinery in distinct membrane domains according to physiological demands [81].

The physiological significance of polarized PMCA localization extends beyond intracellular Ca^2+^ regulation. In transporting epithelia, vectorial Ca^2+^ movement requires coordinated apical entry, intracellular buffering, and basolateral extrusion. Kidney distal tubule cells provide an excellent example. In polarized MDCK cells, PMCA1b and PMCA4b account for approximately one-third of basolateral Ca^2+^ extrusion, working together with the NCX1. Vitamin D_3_ markedly increases PMCA expression, particularly PMCA4b, and simultaneously redistributes the pumps from the apical toward the basolateral membrane. This redistribution significantly enhances apical-to-basolateral Ca^2+^ flux, demonstrating that hormonal regulation controls not only pump abundance but also membrane localization to optimize renal Ca^2+^ reabsorption [82].

Secretory epithelia represent another specialized example of polarized PMCA function. In pancreatic and salivary acinar cells, PMCA is present in both apical and basolateral membranes, although significant enrichment occurs near the luminal pole, where agonist-induced Ca^2+^ signals originate. Surprisingly, despite this polarized localization, PMCA-mediated Ca^2+^ extrusion contributes to relatively uniform cytosolic Ca^2+^ clearance, whereas the initiation and propagation of Ca^2+^ waves depend primarily on polarized SERCA distribution [83].

Disruption of epithelial polarity is a hallmark of numerous human diseases, particularly cancer. Recent work demonstrates that PMCA dysfunction contributes directly to this process rather than merely reflecting loss of epithelial differentiation. This and other studies [84], identified PMCA4 a key regulator of epithelial morphogenesis, linking Ca^2+^ homeostasis to membrane trafficking, cell polarity, and tissue organization. Consequently, loss of PMCA expression may facilitate epithelial-to-mesenchymal transition and tumor progression.

### 6.5. Hypertension and Male Fertility

Accumulating evidence indicates that PMCA1 performs functions extending beyond its classical housekeeping role, particularly in the regulation of cardiovascular physiology and calcium-dependent signaling pathways. Initial evidence for such specialized functions emerged from studies of PMCA4-deficient mice, which developed abnormalities in vascular contractility only when generated on a genetic background harboring a single functional copy of ATP2B1 [85,86]. These observations suggested that PMCA1 acts as a genetic modifier of vascular smooth muscle function and may partially compensate for the loss of other PMCA isoforms. Consistent with this concept, large-scale genome-wide association studies have subsequently identified significant associations between polymorphisms within the ATP2B1 locus and both systolic blood pressure and coronary artery disease in human populations [87,88], implicating PMCA1 as an important determinant of cardiovascular risk. More direct evidence was provided by vascular smooth muscle cell-specific ATP2B1 knockout mice, which exhibited significantly elevated systolic blood pressure accompanied by reduced PMCA1 expression in the aorta. Notably, these animals displayed a compensatory increase in PMCA4 expression; however, this adaptation was insufficient to normalize calcium homeostasis. Vascular smooth muscle cells isolated from these mice showed elevated intracellular Ca^2+^ concentrations under both basal conditions and following stimulation with the vasoconstrictor phenylephrine, indicating that PMCA1 fulfills a nonredundant role in controlling calcium signaling within the vascular wall. Consequently, loss of PMCA1 promotes enhanced vasoconstriction and hypertension through dysregulation of intracellular calcium handling [85,89,90].

PMCA4, in turn, PMCA4 is the predominant plasma membrane Ca^2+^ pump in mature sperm and is essential for maintaining the Ca^2+^ homeostasis required for sperm motility and fertilization. It is highly enriched in the principal piece of the sperm tail, where it rapidly extrudes Ca^2+^ following activation. Genetic ablation of Atp2b4 results in complete male infertility despite normal spermatogenesis and sperm morphology, owing to impaired hyperactivated motility caused by defective Ca^2+^ clearance [91,92]. PMCA4 also contributes to sperm maturation during epididymal transit [93]. These findings establish PMCA4 as an indispensable regulator of sperm maturation, hyperactivated motility, and male fertility.

## 7. PMCA as a Dynamic Regulator of Intracellular Signaling Pathways

Although PMCAs were originally characterized as high-affinity calcium extrusion pumps responsible for maintaining low cytosolic Ca^2+^ concentrations, accumulating evidence indicates that they also function as important organizers of intracellular signaling networks. This regulatory role arises from the ability of PMCAs to interact directly with numerous signaling proteins and to shape highly localized calcium microdomains at the plasma membrane. Consequently, PMCAs influence not only global calcium homeostasis but also the activity of specific calcium-dependent signaling pathways (Table 3).

Many PMCA-interacting proteins identified to date are key components of calcium-sensitive signaling cascades, including calcineurin, neuronal nitric oxide synthase (nNOS), calcineurin/NFAT signaling complexes, protein kinases, and various scaffold proteins. Rather than regulating these proteins through direct enzymatic inhibition, PMCAs appear to modulate their activity by controlling the local calcium concentration in their immediate vicinity. Through continuous calcium extrusion, PMCA generates subplasmalemmal microdomains characterized by exceptionally low Ca^2+^ concentrations. Signaling proteins associated with PMCA are therefore exposed to a calcium environment that differs markedly from the bulk cytosol, resulting in selective suppression of their calcium-dependent activation.

Several lines of evidence support this model. However, it is important to distinguish between calcium microdomains and nanodomains. Calcium microdomains are localized Ca^2+^ signaling regions that have been directly visualized by fluorescence imaging [83,95,96,97,98,99], whereas calcium nanodomains are considerably smaller and currently remain beyond the spatial resolution of conventional optical microscopy. Their existence and functional properties are therefore inferred primarily from indirect experimental observations and computational modeling.

One of the best-characterized examples of such compartmentalized signaling comes from polarized secretory epithelial cells. In pancreatic acinar cells, Ca^2+^ released from the apical endoplasmic reticulum generates a highly localized cytosolic microdomain that drives exocytosis and chloride secretion, while a belt of perigranular mitochondria restricts Ca^2+^ diffusion toward the basal cytoplasm, thereby preserving the spatial specificity of the signal. Simultaneously, the polarized distribution of Ca^2+^ transport systems, including Ca^2+^ release channels, ATP-dependent Ca^2+^ pumps, and mitochondria, establishes vectorial Ca^2+^ fluxes across the cell, coupling basal Ca^2+^ entry and endoplasmic reticulum refilling with apical Ca^2+^ release and extrusion [95]. This organization enables repetitive physiological Ca^2+^ signaling while maintaining stable global cytosolic Ca^2+^ concentrations.

Building on these observations, pioneering studies demonstrated that PMCAs are concentrated within discrete plasma membrane domains rather than being uniformly distributed. Holton and Armesilla subsequently integrated these findings into a functional model proposing that PMCA-enriched membrane regions act as specialized signaling platforms in which continuous Ca^2+^ extrusion generates localized low-Ca^2+^ microdomains that regulate nearby signaling molecules without substantially affecting bulk cytosolic Ca^2+^ concentrations [94]. In polarized epithelial cells, PMCA-rich apical membrane domains therefore function not only as Ca^2+^ extrusion sites but also as organizers of compartmentalized signaling.

**Figure 4:**
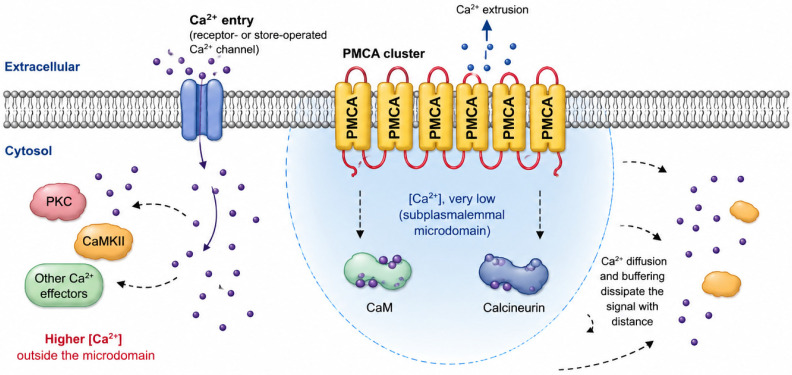
Functional organization of a PMCA-enriched plasma membrane microdomain. PMCA molecules are clustered within specialized plasma membrane domains, such as lipid rafts and caveolae, where continuous Ca^2+^ extrusion generates a localized subplasmalemmal low-Ca^2+^ microdomain. This restricted calcium environment selectively regulates nearby Ca^2+^-dependent signaling proteins, including CaM and calcineurin, while Ca^2+^ concentrations outside the microdomain remain sufficiently elevated to activate other calcium-sensitive effectors. Local Ca^2+^ entry through plasma membrane channels is rapidly buffered by clustered PMCAs, establishing spatially confined calcium gradients without substantially altering global cytosolic Ca^2+^ levels. Diffusion and intracellular buffering progressively dissipate the Ca^2+^ signal with increasing distance from the membrane. The figure illustrates the concept that PMCA functions not only as a high-affinity Ca^2+^ extrusion pump but also as an organizer of compartmentalized calcium signaling by creating localized signaling platforms that control the activity of Ca^2+^-dependent enzymes and downstream signaling pathways.

Consistent with this model, PMCAs are highly enriched within specialized plasma membrane microdomains, particularly caveolae and lipid rafts, rather than being uniformly distributed throughout the membrane [100,101]. In some cell types, PMCA density within caveolar membranes has been reported to be 20–25-fold higher than in the surrounding plasma membrane. Such clustering of active Ca^2+^ pumps creates localized regions of efficient calcium clearance, establishing subplasmalemmal low-Ca^2+^ microdomains in which intracellular Ca^2+^ concentrations remain significantly lower than elsewhere in the cell. As a consequence, Ca^2+^-dependent enzymes residing within these domains experience reduced calcium exposure and exhibit diminished activation (Figure 4). Alternative splicing further contributes to this spatial organization. For example, the PMCA2w splice variant is preferentially targeted to the apical membrane of polarized epithelial cells, whereas PMCA2x localizes predominantly to lateral membrane domains, enabling distinct PMCA variants to regulate Ca^2+^ signaling within specific membrane compartments [81,102].

Direct experimental support for the functional importance of these microdomains was provided by Craske et al., who used GFP-tagged calmodulin to visualize the spatial decoding of intracellular Ca^2+^ signals [103]. Agonist-induced Ca^2+^ elevations rapidly recruited calmodulin to discrete subplasmalemmal secretory microdomains before its subsequent accumulation in the nucleus. Repetitive Ca^2+^ oscillations produced pulsatile calmodulin translocation to the apical secretory microdomain, whereas sustained stimulation promoted progressive nuclear accumulation. These findings provided one of the first direct visual demonstrations that localized Ca^2+^ microdomains not only regulate calmodulin activation but also direct its intracellular redistribution, thereby enabling compartmentalized decoding of Ca^2+^ signals and selective activation of downstream signaling pathways.

Physical association between PMCA and its signaling partners appears to be essential for this regulatory effect. Experimental disruption of PMCA–protein interactions frequently abolishes the inhibitory influence of PMCA on the associated signaling molecule, even though the calcium transport activity of the pump remains fully intact. These observations indicate that bulk calcium extrusion alone is insufficient to regulate signaling pathways. Instead, effective regulation requires the recruitment of signaling proteins into PMCA-generated low-calcium microdomains, where local calcium concentrations are tightly controlled. Thus, spatial organization of signaling complexes is as important as calcium transport itself in determining PMCA-dependent signaling outcomes [4,5,104,105,106,107].

Among the best-characterized examples is the interaction between PMCA4 and neuronal nitric oxide synthase (nNOS) [108]. PMCA4 directly associates with nNOS through PDZ-domain-mediated interactions and suppresses nitric oxide production by maintaining low calcium concentrations around the enzyme. Disruption of this interaction restores nNOS activity despite continued calcium extrusion by PMCA, highlighting the importance of localized calcium signaling. Similar mechanisms have been described for calcineurin, a calcium/calmodulin-dependent phosphatase that controls activation of NFAT transcription factors [109,110]. By restricting local calcium availability, PMCA can attenuate calcineurin activation and thereby regulate calcium-dependent gene transcription. This mechanism is particularly relevant in neurons [111,112], cardiomyocytes [113], immune cells [114], and retinal ganglion cells [115,116,117], where calcineurin/NFAT signaling plays critical roles in differentiation, survival, and stress responses.

PMCA4 has emerged as a particularly important signaling scaffold. In addition to nNOS and calcineurin, PMCA4 interacts with proteins such as CASK, PSD-95, Homer proteins, RASSF family members, and members of the MAGUK family, enabling the assembly of multiprotein signaling complexes at the plasma membrane. Through these interactions, PMCA4 integrates calcium extrusion with diverse signaling pathways involved in cell proliferation, apoptosis, cytoskeletal remodeling, and gene expression [22,91,92,118]. This scaffold function has led to the concept that PMCA4 acts not only as a calcium transporter but also as a signaling hub that coordinates localized cellular responses to calcium signals. Furter studies suggest that PMCA-mediated regulation extends beyond simple suppression of calcium-dependent enzymes [119,120,121]. By dynamically shaping the amplitude, duration, and spatial distribution of calcium signals, PMCAs contribute to the generation of signaling specificity. Two cells experiencing similar global calcium elevations may therefore activate different downstream pathways depending on the local distribution of PMCA isoforms and their associated signaling complexes. This concept is particularly important in excitable tissues, where rapid and highly compartmentalized calcium signaling underlies processes such as neurotransmitter release, synaptic plasticity, muscle contraction, sensory transduction, and neuronal survival.

## 8. PMCA in Cancer

### 8.1. PMCA1

PMCA1, encoded by the ATP2B1 gene, is the most ubiquitously expressed member of the plasma membrane Ca^2+^-ATPase family and has traditionally been regarded as the principal housekeeping calcium pump responsible for maintaining basal intracellular Ca^2+^ homeostasis in virtually all mammalian cell types. Unlike the more tissue-restricted PMCA2 and PMCA3 isoforms, PMCA1 is broadly distributed throughout the body and plays a fundamental role in controlling resting cytosolic calcium concentrations. The physiological importance of this isoform is underscored by the observation that global deletion of ATP2B1 results in early embryonic lethality, demonstrating that PMCA1 is indispensable for embryonic development and organogenesis [91,122]. Despite its essential role in cellular physiology, no specific human disease has been directly attributed to complete loss-of-function mutations in ATP2B1, likely because severe disruption of PMCA1 activity is incompatible with normal development and survival. Nonetheless, recent evidence suggests that ATP2B1 variants may be associated with neurodevelopmental disorders of variable expressivity, and de novo heterozygous mutations and biallelic loss-of-function variants can give rise to severe multisystem phenotypes involving developmental delay, craniofacial dysmorphism, growth impairment, and central nervous system abnormalities [123].

#### 8.1.1. PMCA1 Protein and ATP2B1 Expression in Cancer

Current evidence directly implicating PMCA1 protein in cancer remains relatively limited but suggests context-dependent functions (Table 4).

Early studies demonstrated reduced ATP2B1 expression in oral squamous cell carcinoma compared with normal oral epithelium, supporting the concept that impaired calcium extrusion may contribute to malignant transformation [124]. More broadly, remodeling of calcium transport proteins, including PMCAs, appears to be a common feature of cancer cells, allowing intracellular Ca^2+^ to be maintained within a range that supports proliferation while avoiding calcium-induced cell death [125,126]. PMCA1 was proposed to participate in this remodeling process through its role in maintaining intracellular calcium concentrations within a range that supports proliferation while preventing activation of calcium-dependent cell death pathways. These early studies established the concept that altered PMCA1 expression is not merely a consequence of transformation but may contribute directly to the acquisition of malignant phenotypes.

Further evidence for the role of PMCA1 in cancer progression came from studies of endothelial biology. PMCA1 was shown to be essential for endothelial cell viability, migration, and angiogenic responses. Silencing ATP2B1 in endothelial cells disrupts calcium homeostasis, impairs cell survival and migration, and markedly suppresses angiogenesis, suggesting that PMCA1 may contribute to tumor progression by supporting vascular remodeling [127].

More recent transcriptomic and epigenomic studies further support a role for ATP2B1 expression in cancer biology. In intrahepatic cholangiocarcinoma, higher ATP2B1 expression correlates with lower promoter methylation, increased infiltration of CD4^+^ and CD8^+^ T cells, higher immune and stromal scores, and improved patient survival [128]. These observations were subsequently validated in a large multi-omics analysis that identified ATP2B1 as a prognostic biomarker associated with an immune-active (“hot”) tumor microenvironment and potentially improved responses to immune checkpoint blockade [129]. Similarly, systems biology analyses have identified ATP2B1 among genes associated with breast cancer progression [130]. These findings suggest that PMCA1 may influence cancer progression not only by regulating intracellular calcium homeostasis but also through effects on angiogenesis and the tumor immune microenvironment.

#### 8.1.2. Cancer-Associated Non-Coding Transcripts Derived from the ATP2B1 Locus

In addition to PMCA1 itself, the ATP2B1 genomic locus gives rise to several non-coding transcripts that have independently been implicated in cancer biology. Importantly, the functions of these transcripts should not be interpreted as direct evidence for PMCA1 protein activity or calcium transport.

The antisense long non-coding RNA ATP2B1-AS1 has emerged as a prognostic biomarker in multiple malignancies. In gastric cancer, ATP2B1-AS1 forms part of a pyroptosis-related lncRNA signature associated with overall survival and has been proposed to suppress immune evasion through the miR-425-3p/ZC3H12A signaling axis [131]. Conversely, the circular RNA circATP2B1, also transcribed from the ATP2B1 locus, promotes aerobic glycolysis in gastric cancer cells through regulation of the miR-326 pathway [132]. ATP2B1-AS1 has likewise been associated with tumor progression or prognosis in lung adenocarcinoma [133], esophageal squamous cell carcinoma [134], and colorectal cancer [135,136], where it forms part of prognostic lncRNA signatures and has been linked to immune regulation and therapeutic response. Although these observations clearly indicate that the ATP2B1 genomic locus contributes to cancer biology through multiple regulatory mechanisms, the available evidence does not demonstrate that these effects are mediated by PMCA1 protein or its calcium transport activity. Rather, they highlight the complexity of the ATP2B1 locus, which encodes both the PMCA1 calcium pump and several independently functioning regulatory non-coding RNAs.

Finally, recent work identifying ATP2B1 as a marker of human hematopoietic stem cells with superior repopulating capacity further supports a role for ATP2B1-associated signaling in stem-like cellular states relevant to tumor initiation and therapy resistance [137]. However, the embryonic lethality of constitutive Atp2b1 knockout mice has substantially limited mechanistic investigations of PMCA1 in cancer using conventional in vivo models. Consequently, elucidating the context-dependent functions of PMCA1 during tumor initiation and progression will require tissue-specific and inducible genetic models that circumvent its essential role in embryonic development. Future studies should therefore distinguish PMCA1-dependent calcium transport from regulatory mechanisms mediated by ATP2B1-derived non-coding RNAs to clarify their respective contributions to tumor progression.

### 8.2. PMCA2

PMCA2, encoded by the ATP2B2 gene, is a high-affinity calcium extrusion pump best known for its physiological roles in excitable tissues and in the lactating mammary gland. Among the four PMCA isoforms, PMCA2 is distinguished by its tissue-selective expression pattern, rapid activation kinetics, and particularly high basal activity, properties that make it well suited for environments characterized by intense or highly localized calcium fluxes [106,111]. Although this isoform was originally studied in the context of hearing, balance, and mammary calcium transport during lactation [138], the biological rationale for implicating PMCA2 in cancer is straightforward.

The strongest evidence linking PMCA2 to cancer comes from breast cancer. Early studies demonstrated that PMCA2 expression is retained or re-expressed in a subset of breast tumors. Initial comparisons of human breast epithelial cell lines showed that PMCA2 mRNA can be markedly elevated in certain tumorigenic breast cancer lines relative to non-tumorigenic mammary epithelial cells. In some cases, ATP2B2 expression was reported to be increased by more than two orders of magnitude, indicating that PMCA2 overexpression is not a subtle phenomenon but can represent a major remodeling event in the calcium extrusion machinery of malignant cells [139,140].

Subsequent immunohistochemical and transcriptomic analyses strengthened this concept [140]. Although distinct membrane staining was observed in only approximately 9% of tumors, transcriptomic analyses demonstrated that ATP2B2 expression is enriched in basal-like breast cancers, suggesting that PMCA2 expression is subtype-dependent and may be regulated at multiple levels. The same authors demonstrated that silencing of PMCA2 reduced MDA-MB-231 cell proliferation, decreased the proportion of cells entering S phase, and sensitized cells to doxorubicin. Notably, analogous silencing of PMCA1 or PMCA4 in the same system did not reproduce this antiproliferative effect, arguing that PMCA2 performs a nonredundant role that cannot be simply compensated by other PMCA family members. At first glance, these findings appear inconsistent with those of Curry and colleagues [141], who reported that siRNA-mediated PMCA2 silencing alone had little effect on the viability of MDA-MB-231 cells. However, it significantly sensitized these cells to calcium-dependent apoptosis induced by ionomycin and potentiated cell death triggered by the Bcl-2 inhibitor ABT-263 (Navitoclax). Rather than being contradictory, these studies examine distinct biological endpoints and together support a context-dependent model of PMCA2 function. Peters and colleagues focused on cell proliferation and cell-cycle progression under basal growth conditions, whereas Curry et al. investigated apoptotic susceptibility under pharmacological stress. Collectively, their findings indicate that PMCA2 fulfills complementary roles depending on the cellular context: under physiological growth conditions, it maintains intracellular calcium homeostasis required for cell-cycle progression and proliferation, whereas during cellular stress it acts primarily as an anti-apoptotic regulator that limits calcium overload and enhances resistance to cell death. Thus, PMCA2 appears to promote both tumor growth and survival, highlighting its potential as a therapeutic target whose inhibition could simultaneously suppress proliferation and sensitize breast cancer cells to anticancer therapy.

The relationship between PMCA2 and apoptosis has even deeper roots in mammary biology. During lactation, PMCA2 is highly expressed at the apical membrane of mammary epithelial cells, where it mediates calcium transport into milk under the control of the calcium-sensing receptor (CaSR). Activation of the CaSR simultaneously stimulates PMCA2-dependent calcium transport while suppressing parathyroid hormone-related protein (PTHrP) production, thereby coordinating mammary calcium secretion with maternal calcium homeostasis. Remarkably, malignant transformation fundamentally alters this regulatory circuit. Although breast cancer cells retain CaSR expression, CaSR signaling becomes rewired to stimulate PTHrP production while maintaining high PMCA2 expression. This switch transforms a physiological feedback mechanism into a feed-forward pathway that promotes tumor progression and contributes to the development of osteolytic bone metastases [142,143]. VanHouten and colleagues further showed that PMCA2 expression rapidly declines after weaning, leading to increased intracellular Ca^2+^ concentrations and activation of mammary epithelial apoptosis. Conversely, sustained PMCA2 expression protected mammary epithelial cells from calcium-induced cell death. Immunohistochemical analysis of human breast cancer samples revealed that high PMCA2 expression was associated with significantly poorer patient survival, suggesting that breast tumors exploit PMCA2 to evade apoptosis by maintaining low intracellular Ca^2+^ concentrations [78].

Jeong et al. [144] provided compelling evidence that PMCA2 functions as a key regulator of HER2-driven breast cancer. PMCA2 expression strongly correlates with HER2 levels in human breast tumors and MMTV-Neu mouse models, and high co-expression of both proteins is associated with significantly poorer patient survival. PMCA2 colocalizes with HER2 in specialized plasma membrane microdomains, where it maintains a localized low-calcium environment that stabilizes the receptor. Knockdown of PMCA2 in HER2-positive breast cancer cells reduces HER2 expression and phosphorylation, suppresses EGFR/HER3/AKT signaling, disrupts HER2-HSP90 interactions, and promotes receptor ubiquitination and lysosomal degradation. Consistent with these findings, genetic deletion of ATP2B2 in MMTV-Neu mice markedly delays mammary tumor development and reduces HER2 signaling, demonstrating that PMCA2 is an essential facilitator of HER2 stability and oncogenic signaling rather than merely a marker of HER2-positive breast cancer. The importance of sustained EGFR-family signaling in therapeutic resistance is further illustrated by recent studies showing that YY1 promotes cetuximab resistance in KRAS-mutant colorectal cancer through persistent activation of the KRAS–EGFR–AKT/ERK signaling axis [145].

Subsequent reports demonstrated that HER2 and PMCA2 participate in a reciprocal regulatory network. HER2 signaling itself maintains the actin-rich membrane protrusions in which HER2, PMCA2, HSP90, and additional signaling proteins are concentrated. Partial knockdown of HER2 expression or pharmacological inhibition with lapatinib disrupted these membrane structures, increased intracellular calcium concentrations, reduced PMCA2 expression, and promoted HER2 ubiquitination and degradation. These findings suggest the existence of a positive feedback loop in which active HER2 signaling preserves PMCA2-containing membrane nanodomains, while PMCA2 appears to support the integrity of HER2-associated signaling complexes at the cell surface, thereby reinforcing sustained receptor activation [146].

Further mechanistic studies identified the scaffolding protein NHERF1 as another essential component of this signaling complex. NHERF1 binds directly to the PDZ-binding motif located within the C-terminal tail of PMCA2 and forms a multiprotein complex containing PMCA2, HER2, and HSP90 within actin-rich membrane domains. NHERF1 expression was significantly increased in HER2-positive tumors, correlated with PMCA2 expression, and was associated with HER2-positive status in both ductal carcinoma in situ and invasive breast cancers. Silencing NHERF1 reduced PMCA2 expression, disrupted HER2–HSP90 interactions, inhibited HER2 signaling, and accelerated receptor internalization and degradation [147]. The stabilization of this membrane complex is provided by ezrin, which expression is markedly elevated in HER2-positive breast cancers. Pharmacological inhibition or genetic depletion of ezrin disrupted the entire HER2–PMCA2–NHERF1 complex, promoted PKC-dependent HER2 internalization and degradation, and markedly inhibited HER2 signaling. Importantly, ezrin inhibition acted synergistically with lapatinib to suppress proliferation and induce apoptosis in HER2-positive breast cancer cells, suggesting that destabilization of PMCA2-containing membrane signaling complexes may enhance the therapeutic efficacy of HER2-targeted treatments [148]. Recent work has further expanded this model by identifying the polarity protein Erbin as an additional component of the PMCA2-dependent HER2 signaling complex. In normal mammary epithelial cells, Erbin localizes to the basolateral membrane, whereas PMCA2, NHERF1, and ezrin are confined to the apical membrane. During early HER2-driven tumorigenesis, disruption of apical-basal polarity allows these proteins to redistribute and assemble into a multiprotein PMCA2–NHERF1–ezrin–HER2–Erbin complex within actin-rich membrane protrusions. Analyses of MMTV-Neu mice, human HER2-positive ductal carcinoma in situ, and spatial transcriptomic datasets demonstrated that formation of this complex is an early event during malignant transformation and is accompanied by coordinated upregulation of ERBIN, EZR, SLC9A3R1 together with suppression of genes involved in apical junction organization [149].

Recent evidence also suggests that PMCA2 may contribute to tumor-associated stromal remodeling [150]. Transcriptomic analyses of malignant breast calcifications identified calcium homeostasis pathways among the major molecular changes accompanying stromal osteogenic differentiation. Functional studies demonstrated that overexpression of squalene epoxidase (SQLE) in adipose-derived mesenchymal stem cells induced early upregulation of PMCA2 together with the mitochondrial calcium uniporter, preceding osteogenic differentiation and sustained mitochondrial calcium accumulation. In xenograft models, SQLE-overexpressing stromal cells promoted ectopic calcification within breast tumors, suggesting that PMCA2 participates in calcium remodeling of the tumor microenvironment rather than acting exclusively within malignant epithelial cells.

Clinical evidence further supports the importance of PMCA2 alternative splicing in breast cancer [151]. Analysis of tumor specimens from 85 breast cancer patients demonstrated that multiple ATP2B2 splice variants are expressed in both tumor and adjacent normal tissues, although their abundance differs according to tumor subtype. Among the analyzed isoforms, PMCA2b showed the strongest clinical association, being significantly overexpressed in HER2-positive tumors compared with HER2-negative cancers. In contrast, PMCA2w expression correlated with estrogen receptor positivity, whereas PMCA2z expression was associated with progesterone receptor-positive tumors. Moreover, PMCA2x/b expression was significantly higher in lobular than ductal carcinomas, indicating that alternative splicing contributes to the molecular heterogeneity of breast cancer.

A second, more nuanced mechanism may involve the generation of localized calcium microdomains rather than simply reducing global cytosolic Ca^2+^ levels. In addition, PMCA2 appears to function as a component of membrane-associated signaling scaffolds that regulate downstream signaling pathways independently of its calcium extrusion activity. The first evidence linking PMCA2 to oncogenic signaling came from Holton et al., who demonstrated that PMCA2 directly interacts with the calcium/calmodulin-dependent phosphatase calcineurin in breast cancer cells. By recruiting calcineurin into PMCA-generated low-calcium microdomains, PMCA2 suppresses calcineurin phosphatase activity and reduces activation of the NFAT transcription factors [109]. Similar regulation of the calcineurin/NFAT pathway has also been demonstrated in pheochromocytoma-derived PC12 cells, where PMCA2 knockdown increased calcineurin activity, promoted NFAT nuclear translocation, and altered the expression of numerous calcium-responsive genes involved in neuronal differentiation and secretory function [45,110,152,153]. Further mechanistic insight was provided by Baggott et al., who selectively disrupted the interaction between PMCA2 and calcineurin using competitive peptides. Dissociation of the PMCA2–calcineurin complex restored calcineurin activity, increased NFAT activation, induced Fas ligand expression, and significantly enhanced apoptosis in multiple breast cancer cell lines. Moreover, disruption of this interaction markedly increased the cytotoxic effects of paclitaxel, demonstrating that PMCA2-mediated inhibition of calcineurin contributes directly to chemotherapy resistance [154].

A completely new dimension of PMCA2 biology has recently emerged from studies investigating cytocapsulas and cytocapsular tubes, specialized membrane structures surrounding malignant cells [155]. Proteomic analyses of isolated cytocapsular membranes from pancreatic, breast, and colorectal cancer cells identified PMCA2 as one of the most abundant proteins within these structures. Immunohistochemical analyses demonstrated minimal PMCA2 expression in normal tissues and benign tumors but strong enrichment in cytocapsular membranes across numerous human malignancies. The authors further proposed that PMCA2-enriched cytocapsular tubes provide protected migration routes that promote tumor spread, highlighting the pump as a potential therapeutic target for limiting metastasis.

Overall, the current literature supports a model in which PMCA2 functions as a cancer-relevant calcium efflux pump that is particularly important in breast cancer. PMCA2 is overexpressed in a subset of tumors, enriched in basal-like breast cancers, and functionally required for optimal proliferation and drug resistance in experimental systems. These effects are biologically plausible given the normal role of PMCA2 in mammary epithelial calcium transport and survival, as well as its kinetic suitability for protecting cells from calcium overload. At the same time, PMCA2 should not be viewed simply as a generic anti-apoptotic pump. Its effects likely depend on tumor subtype, splice-variant composition, membrane localization, and the balance between calcium signals that promote proliferation and those that trigger death. The diverse, context-dependent functions of PMCA2 described not only in breast cancer but also in other malignancies, including its roles in regulating cell proliferation, apoptosis, HER2 signaling, and calcineurin/NFAT signaling are summarized in Table 5.

### 8.3. PMCA3

PMCA3 (ATP2B3) exhibits a highly restricted distribution and is especially abundant in cerebellar Purkinje neurons, where rapid and precise regulation of intracellular Ca^2+^ is essential for neuronal excitability, synaptic transmission, and motor coordination. Like PMCA2, PMCA3 displays fast activation kinetics and high affinity for Ca^2+^, making it particularly well suited for shaping transient subplasmalemmal Ca^2+^ signals generated during repetitive neuronal activity [156]. Although only a limited number of animal models have been generated to investigate PMCA3 function, the available evidence indicates that this isoform plays an important role in the regulation of neuronal calcium signaling. Atp2b3 knockout mice, generated using a triple-target CRISPR/Cas9 approach, exhibited a reproducible long-sleeper phenotype supporting the concept that PMCA3-mediated Ca^2+^ extrusion contributes to the control of neuronal excitability through Ca^2+^-dependent hyperpolarization pathways [157]. Most studies have focused on its function in neuronal physiology and hereditary neurological disorders, whereas evidence linking PMCA3 directly to tumorigenesis is relatively limited. Nevertheless, emerging genomic and molecular data suggest that alterations in ATP2B3 may contribute to specific cancer types through dysregulation of intracellular Ca^2+^ signaling.

The strongest association between PMCA3 and cancer has been described in aldosterone-producing adrenal adenomas (APAs). Although APAs are not malignant, they represent the first and currently best-established example in which ATP2B3 mutations have been recurrently identified across independent cohorts of aldosterone-producing adenomas, although they occur at a relatively low frequency compared with the major driver genes KCNJ5, CACNA1D, ATP1A1, and CTNNB1. These somatic ATP2B3 mutations were first identified by exome sequencing together with mutations in KCNJ5, ATP1A1, and CACNA1D, revealing that abnormalities in ion transport constitute a common molecular mechanism underlying APA development. ATP2B3 mutations account for approximately 1–5% of sporadic APAs, although their prevalence varies among patient cohorts and ethnic populations. Large sequencing studies from Europe and the United States consistently reported ATP2B3 mutations in approximately 4% of CYP11B2 (aldosterone synthase)—positive adenomas, while similar frequencies were observed in Black American cohorts, indicating that ATP2B3 mutations represent a relatively uncommon but recurrent genetic event in APA pathogenesis [158,159,160]. More recently, analysis of a Brazilian cohort identified ATP2B3 variants in 11.3% of CYP11B2-positive lesions, representing a significantly higher prevalence than previously reported and suggesting population-specific differences in the genetic architecture of primary aldosteronism. In that study, ATP2B3-mutated tumors occurred predominantly in older male patients [161,162,163]. Transcriptomic studies similarly demonstrated that ATP2B3-mutant tumors cluster with ATP1A1-mutant adenomas and exhibit gene expression profiles distinct from CTNNB1-mutant tumors, further supporting the existence of molecularly defined APA subgroups [164,165]. Interestingly, ATP2B3 mutations are considerably less frequent than mutations in KCNJ5, CACNA1D, or ATP1A1 and occur in both posture-responsive and posture-unresponsive APAs, although only a small number of ATP2B3-mutated tumors have been identified in these cohorts. Nevertheless, ATP2B3 belongs to the established spectrum of recurrent aldosterone-driver mutations that collectively account for more than 90% of CYP11B2-positive APAs when modern immunohistochemistry-guided sequencing approaches are used [166,167].

Unlike KCNJ5 mutations, which depolarize adrenal cells through abnormal sodium conductance, ATP2B3 mutations directly disrupt calcium homeostasis, demonstrating that multiple genetic alterations converge on intracellular Ca^2+^ signaling as the central mechanism driving hormone hypersecretion in APAs. Although these mutations clearly explain autonomous aldosterone production, their contribution to tumor cell proliferation remains less well defined, and current evidence suggests that additional genetic or microenvironmental events may be required for adenoma formation [160,168,169].

Histopathological analyses further indicate that ATP2B3-mutant adenomas represent a distinct biological subtype. In contrast to the larger KCNJ5-mutated tumors, ATP2B3-mutant APAs are generally smaller and display a zona glomerulosa-like phenotype. They also exhibit relatively higher Ki67 labeling than KCNJ5-mutant tumors, suggesting subtle differences in proliferative activity despite their overall benign behavior. Morphometric analyses demonstrated that ATP2B3-mutated tumors are generally enriched in clear, lipid-rich tumor cells, resembling KCNJ5-mutated adenomas but displaying distinct patterns of steroidogenic differentiation. Comparative analyses of cholesterol metabolism revealed that ATP2B3-mutated tumors belong to the non-KCNJ5 subgroup, exhibiting cholesterol metabolic characteristics distinct from KCNJ5-mutated lesions, highlighting that different aldosterone-driver mutations generate unique metabolic phenotypes despite converging on intracellular calcium signaling [170].

The biological consequences of ATP2B3 mutations also include altered endocrine responsiveness. Comparative transcriptomic analyses demonstrated that aldosterone-producing adenomas harboring ATPase mutations (ATP2B3 and ATP1A1) exhibit distinct expression profiles of ACTH receptor components compared with KCNJ5-mutated tumors, suggesting that ATPase-mutated adenomas retain greater sensitivity to ACTH stimulation and therefore differ in the hormonal regulation of aldosterone secretion [171]. The functional importance of ATP2B3 mutations has been further confirmed in comparative studies of other PMCA family members. Functional analyses of PMCA4 variants employed the previously characterized ATP2B3 mutation as a positive control and confirmed that pathogenic ATP2B3 variants increase CYP11B2 expression, consistent with enhanced calcium-dependent aldosterone synthesis. In contrast, ATP2B4 variants failed to reproduce this phenotype, emphasizing the specific pathogenic role of ATP2B3 dysfunction in adrenal endocrine tumorigenesis [172].

Outside adrenal tumors, the role of ATP2B3 in cancer remains poorly understood, with current evidence deriving largely from transcriptomic and bioinformatic analyses rather than direct mechanistic investigations. In glioma, integrated analyses of TCGA and GEO datasets identified ATP2B3 as one of three prognostic biomarkers significantly associated with overall survival, disease-specific survival, and progression-free survival. ATP2B3 expression also correlated with clinicopathological characteristics, immune cell infiltration, and predicted sensitivity to several anticancer agents. Experimental validation further demonstrated reduced ATP2B3 expression in glioma tissues compared with normal brain, while functional studies suggested that ATP2B3 downregulation promotes glioma cell proliferation and migration, supporting a potential tumor-suppressive role in this malignancy [173]. Similarly, in kidney renal clear cell carcinoma, ATP2B3 was identified as a component of a seven-gene prognostic signature predictive of patient survival. However, ATP2B3 was neither found to be recurrently mutated nor functionally validated as a driver of renal carcinogenesis, indicating that its prognostic value currently relies solely on transcriptomic associations. Collectively, these studies suggest that, outside endocrine tumors, ATP2B3 may serve as a useful prognostic biomarker in selected cancers, although convincing mechanistic evidence supporting a direct role in malignant transformation or tumor progression remains lacking [174].

### 8.4. PMCA4

Among the four PMCA isoforms, PMCA4 (ATP2B4) is the best-characterized in the context of cancer and has emerged as a key regulator of tumor-associated calcium signaling. PMCA4 is ubiquitously expressed and is distinguished by its broad tissue distribution, relatively slow activation kinetics, and extensive interactome, enabling it to regulate both global calcium homeostasis and highly compartmentalized calcium microdomains. Unlike PMCA1, which is essential for embryonic development, PMCA4-deficient mice are viable and develop normally. However, these animals exhibit several tissue-specific phenotypes that underscore its specialized physiological functions. The most striking phenotype is complete male infertility, whereas female fertility remains unaffected [22,175]. In addition, PMCA4 deficiency disrupts calcium-dependent signaling in B lymphocytes through altered interactions with the inhibitory receptor CD22 and the tyrosine phosphatase SHP-1 [176]. Studies of bladder smooth muscle further demonstrate that PMCA4 and PMCA1 fulfill distinct physiological roles. Whereas PMCA1 primarily mediates global cytosolic Ca^2+^ clearance, PMCA4 is specifically required for agonist-induced contractile responses, such as carbachol-stimulated contraction [177]. Collectively, these observations indicate that, unlike the housekeeping isoform PMCA1, PMCA4 primarily fine-tunes localized calcium signaling and stimulus-dependent cellular responses rather than maintaining basal intracellular calcium homeostasis.

The role of PMCA4 in cancer, however, is highly context dependent and varies considerably among different tumor types. One of the earliest observations linking PMCA4 to tumor suppression came from studies of cancer cell differentiation. In both breast and colorectal carcinoma models, PMCA4 expression was markedly reduced compared with normal epithelial tissues but increased during differentiation induced by histone deacetylase inhibitors, short-chain fatty acids, post-confluent growth, or phorbol ester treatment. In MCF-7 breast cancer cells, differentiation selectively and robustly upregulated the PMCA4b splice variant without significantly affecting other PMCA isoforms. Similarly, in Caco-2 colon cancer cells, PMCA4b expression increased during spontaneous differentiation, whereas vitamin D_3_ selectively induced PMCA1b but not PMCA4b [178,179].

Aung et al. demonstrated that PMCA4 expression is reduced in colorectal tumors compared with normal colonic epithelium, a finding confirmed in both matched patient samples and a large microarray dataset. Consistently, differentiation of HT-29 cells induced PMCA4 upregulation and plasma membrane localization, whereas PMCA4 overexpression suppressed cell proliferation and FOS expression, while PMCA4 knockdown enhanced agonist-induced Ca^2+^ transients without affecting apoptosis or TRAIL sensitivity. These findings suggest that loss of PMCA4 primarily promotes proliferative Ca^2+^ signaling rather than resistance to cell death [180]. In contrast, Geyik et al. found no significant differences in total ATP2B4 mRNA expression between colorectal tumors and matched normal tissues, except for a modest increase in rectal tumors detected by semi-quantitative PCR, indicating that total ATP2B4 expression is not consistently altered in colorectal cancer [181]. The apparent discrepancy between these studies can largely be explained by differences in experimental design and analytical approach. Aung et al. combined mechanistic analyses in colon cancer cell lines with validation in a large independent clinical microarray dataset, providing evidence that loss of PMCA4 is associated with dedifferentiation and contributes to calcium signaling remodeling that favors proliferation. In contrast, Geyik et al. examined a relatively small and clinically heterogeneous patient cohort using measurements of total ATP2B4 transcripts without assessing PMCA4 protein levels, splice variants or differentiation status. Consequently, subtle, differentiation-dependent alterations in PMCA4 expression may have been obscured by tumor heterogeneity and the inability to distinguish individual PMCA4 isoforms.

Recent analysis of more than one hundred hormone receptor-positive breast cancer specimens demonstrated a marked reduction in PMCA4 protein expression compared with normal mammary epithelium [84]. Bioinformatic analyses further showed that low ATP2B4 expression was associated with shorter relapse-free survival in patients with luminal A and luminal B1 breast cancers. Functional studies using MCF-7 cells revealed that PMCA4 silencing disrupted epithelial organization by inducing internalization of E-cadherin, impairing apical-basal polarity, and preventing lumen formation in three-dimensional cultures. These effects depended on Arf6-mediated recycling of PMCA4b to the plasma membrane, while silencing the single PMCA orthologue in *Drosophila melanogaster* similarly disrupted salivary gland lumen morphology, indicating that the role of PMCA4 in epithelial morphogenesis is evolutionarily conserved.

A similar tumor-suppressive function has been demonstrated in melanoma. Initial studies demonstrated that PMCA4b expression is markedly reduced in BRAF-mutant melanoma cells and human melanoma specimens compared with benign melanocytic nevi. Pharmacological inhibition of the MAPK pathway using either the BRAF inhibitor vemurafenib or the MEK inhibitor selumetinib restored PMCA4b expression, accelerated cytosolic Ca^2+^ clearance following store-operated Ca^2+^ entry, significantly inhibited melanoma cell migration, and markedly reduced pulmonary metastasis formation in vivo, establishing PMCA4b as a metastasis suppressor [182]. These findings were subsequently extended by demonstrating that the histone deacetylase inhibitors SAHA (vorinostat) and valproic acid also induced PMCA4b expression, while further potentiating the effect of vemurafenib. Restoration of PMCA4b was consistently accompanied by reduced migratory capacity, suggesting that epigenetic regulation of ATP2B4 contributes to the anti-metastatic activity of HDAC inhibitors [183].

The clinical relevance of these experimental observations was confirmed in a subsequent study analyzing human melanoma specimens [184]. PMCA4 was detected in both primary and metastatic melanomas, with plasma membrane localization varying according to the degree of tumor differentiation. Moreover, high ATP2B4 transcript levels were associated with significantly longer progression-free survival in female patients with stage I–III melanoma and with improved overall survival in patients receiving anti-PD-1 immune checkpoint therapy. Although PMCA4 protein expression in resected lung metastases correlated primarily with tumor differentiation rather than survival after metastasectomy, these findings strongly support the concept that PMCA4 functions as a metastasis suppressor in melanoma and further suggest that ATP2B4 expression may serve as a predictive biomarker for response to immune checkpoint inhibition.

An interesting contrasting observation was reported by Bahrami et al., who identified a novel ATP2B4–PRKCA fusion in a rare congenital pigment-synthesizing melanocytic neoplasm [185]. However, this rearrangement does not indicate an oncogenic role for PMCA4. Instead, ATP2B4 served as the fusion partner driving aberrant expression of the constitutively active PRKCA kinase domain, while the normal calcium pump was disrupted. Accordingly, the pathogenic effect was attributed to PRKCA activation rather than to PMCA4 function.

A markedly different role of PMCA4 has been described in pancreatic ductal adenocarcinoma (PDAC)**,** where the calcium pump appears to promote several hallmarks of tumor progression. Transcriptomic analysis of the Oncomine database demonstrated that ATP2B4 was the predominantly upregulated PMCA isoform in PDAC, exhibiting a 2.65-fold increase compared with matched normal pancreatic tissue, whereas ATP2B1 was only modestly elevated and ATP2B2 and ATP2B3 were significantly downregulated. Analysis of the TCGA cohorts further revealed that high ATP2B4 expression was associated with significantly reduced overall survival while ATP2B1 expression showed no prognostic significance, suggesting a specific association between PMCA4 overexpression and aggressive disease [118]. Functional evidence linking impaired PMCA activity to pancreatic tumorigenesis was recently provided using the physiologically relevant Kras^G12D^/Trp53^R172H^ KPC mouse model of PDAC. By selectively isolating plasma membrane Ca^2+^ extrusion after SERCA inhibition and extracellular Ca^2+^ removal, the authors demonstrated that cytosolic Ca^2+^ clearance was significantly slower in premalignant acinar cells from KPC mice than in control cells, despite unchanged PMCA expression. These findings indicate that impaired PMCA-mediated Ca^2+^ extrusion is an early functional alteration during pancreatic carcinogenesis and is likely associated with reduced ATP availability rather than decreased pump abundance [186].

PMCA4 has also been implicated in the development of chemotherapy resistance. In epithelial ovarian cancer, comparative analyses of parental MDAH-2774 cells and a cisplatin-resistant derivative generated by stepwise drug selection demonstrated extensive reprogramming of calcium signaling, characterized by significantly lower intracellular Ca^2+^ concentrations and coordinated downregulation of multiple calcium-handling proteins, including PMCA4, SERCA isoforms, IP_3_ receptors, ryanodine receptors, and sodium-calcium exchangers [187]. Similarly, studies in parental K562 leukemia cells and their doxorubicin-resistant counterpart showed that alterations in iron metabolism were accompanied by differential regulation of PMCA isoforms, including PMCA4, together with marked changes in the expression of IP_3_ receptors, SERCA pumps, and other calcium transport proteins. Iron-dependent modulation of these calcium-handling molecules further emphasized the close relationship between metabolic adaptation and calcium signaling during the acquisition of multidrug resistance [188]. Although neither study established a direct causal role for PMCA4 in conferring chemoresistance, both consistently demonstrate that PMCA4 forms part of a broader adaptive remodeling of calcium regulatory networks that accompanies the resistant phenotype. These findings support the concept that disruption of calcium homeostasis is a common feature of chemotherapy adaptation and identify PMCA4, together with other calcium transport systems, as a potential target for therapeutic strategies aimed at overcoming drug resistance.

Immunohistochemical analysis of 318 gastric cancer specimens demonstrated that low PMCA4 expression correlated with advanced tumor-node-metastasis stage and poor patient prognosis. Functional studies in MKN45 and NCI-N87 gastric cancer cells showed that siRNA-mediated PMCA4 silencing induced a pronounced epithelial-to-mesenchymal transition characterized by reduced expression of E-cadherin, increased vimentin expression, and acquisition of an elongated fibroblast-like morphology [189]. Mechanistically, PMCA4 depletion increased intracellular Ca^2+^, promoted nuclear translocation of NFATc1, and induced expression of the EMT transcription factor ZEB1. Genetic silencing of either NFATc1 or ZEB1, as well as pharmacological inhibition of calcineurin with cyclosporine A, abolished the EMT phenotype induced by PMCA4 loss, establishing the PMCA4–NFATc1–ZEB1 axis as a major mechanism by which PMCA4 suppresses gastric cancer metastasis [189].

**Figure 5:**
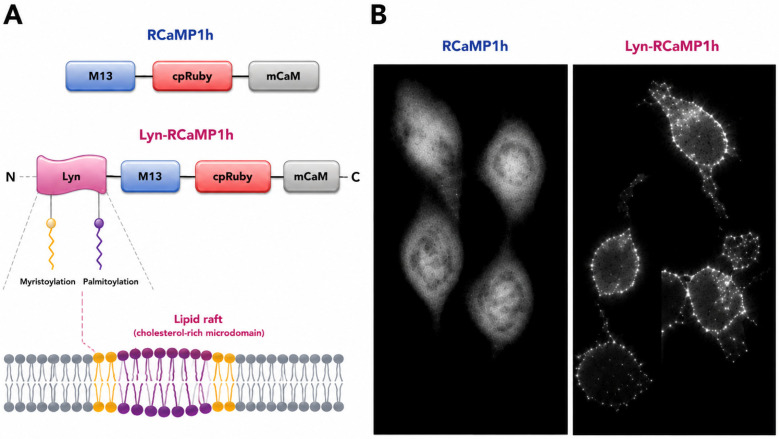
Plasma membrane lipid raft-targeted RCaMP1h biosensor for compartmentalized Ca^2+^ imaging. (**A**) Schematic representation of the genetically encoded intensiometric calcium sensors RCaMP1h and Lyn-RCaMP1h. RCaMP1h consists of the M13 peptide, circularly permuted Ruby fluorescent protein (cpRuby), and calmodulin (mCaM). In Lyn-RCaMP1h, the N-terminal lipid modification sequence derived from Lyn kinase directs the sensor to cholesterol-rich plasma membrane lipid rafts through myristoylation and palmitoylation, enabling selective monitoring of Ca^2+^ dynamics within this membrane microdomain. (**B**) Representative grayscale fluorescence images of glioma cells expressing cytosolic RCaMP1h or lipid raft-targeted Lyn-RCaMP1h. While RCaMP1h is diffusely distributed throughout the cytoplasm, Lyn-RCaMP1h shows preferential localization at the plasma membrane, consistent with targeting to lipid raft microdomains.

Recent evidence indicates that PMCA4 contributes to glioma progression by organizing compartmentalized Ca^2+^ signaling within specialized plasma membrane microdomains rather than simply regulating bulk cytosolic Ca^2+^ homeostasis [190]. PMCA4 forms a functional complex with the GABA transporter GAT3 in cholesterol-rich lipid rafts, where it precisely controls both resting and stimulus-evoked local Ca^2+^ dynamics. Using genetically encoded Ca^2+^ biosensors specifically targeted to lipid rafts, recent live-cell imaging studies directly demonstrated that PMCA4 selectively regulates Ca^2+^ signals within these membrane microdomains (Figure 5).

**Figure 6:**
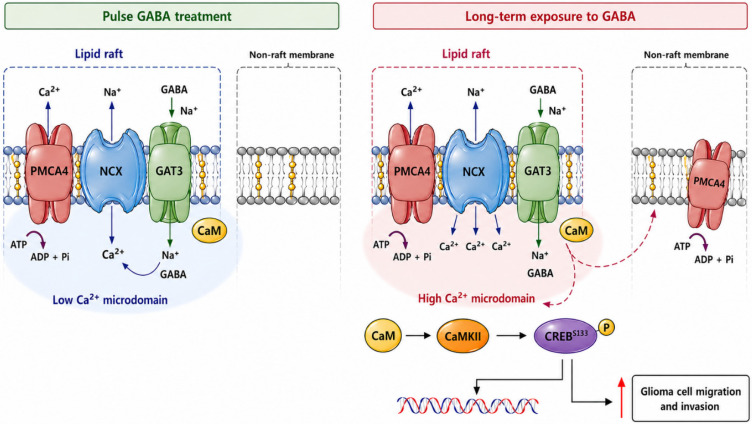
PMCA4 controls compartmentalized Ca^2+^ signaling through dynamic association with the GAT3–NCX complex in glioma lipid rafts. During transient GABA stimulation, PMCA4, NCX, and GAT3 remain closely associated within lipid rafts, where PMCA4 rapidly removes Ca^2+^ entering through NCX, generating a low-Ca^2+^ microdomain that restrains Ca^2+^/CaM-dependent signaling. Sustained GABA exposure disrupts this complex and promotes PMCA4 redistribution to non-raft membrane regions. Consequently, local Ca^2+^ accumulates within lipid rafts, activating the CaM/CaMKII/CREB signaling axis and reducing glioma cell migration and invasion.

Silencing PMCA4 increased both basal and GABA-induced Ca^2+^ accumulation specifically in lipid rafts without proportionally affecting global cytosolic Ca^2+^ levels. Disruption of the PMCA4–GAT3 complex impaired glioma cell migration and invasion through activation of the Ca^2+^/calmodulin-dependent CaMKII/CREB signaling pathway. Importantly, selective buffering of lipid raft-associated Ca^2+^ by targeted expression of raft-localized parvalbumin restored the migratory phenotype, providing direct functional evidence that PMCA4 regulates glioma invasiveness through spatially restricted Ca^2+^ microdomains rather than global calcium homeostasis (Figure 6). These findings represent one of the clearest demonstrations that PMCA4 functions as an organizer of localized Ca^2+^ signaling platforms that selectively control downstream oncogenic signaling pathways.

A complementary mechanism was identified in a subsequent study investigating acylphosphatase 2 (ACYP2), which physically interacts with PMCA4 and enhances PMCA4-mediated Ca^2+^ extrusion. ACYP2 overexpression was associated with increased c-Myc and STAT3 activity together with enhanced glioma cell proliferation, migration, invasion, colony formation, and tumor growth in xenograft models. Importantly, PMCA4 silencing or pharmacological elevation of intracellular Ca^2+^ abolished these ACYP2-dependent phenotypes, indicating that PMCA4 is required for ACYP2-mediated oncogenic signaling. Although these findings support a functional ACYP2–PMCA4–c-Myc–STAT3 signaling axis, the intermediate molecular events linking altered Ca^2+^ homeostasis to transcription factor activation remain incompletely defined, and the temporal sequence of pathway activation has yet to be established [191].

Besides its function as a calcium pump, PMCA4 also participates in the activity regulation of membrane-associated signaling complexes. Previous work demonstrated that PMCA4 directly binds the phosphatase calcineurin, recruiting it into PMCA-generated low-calcium microdomains where calcineurin activity is suppressed [109]. Baggott and colleagues extended this mechanism to endothelial cells and demonstrated that PMCA4 negatively regulates VEGF-induced angiogenesis. Silencing PMCA4 enhanced calcineurin/NFAT signaling, increased expression of the NFAT target genes RCAN1.4 and COX-2, stimulated endothelial migration, and promoted capillary tube formation. Conversely, PMCA4 overexpression inhibited VEGF-dependent NFAT activation and markedly reduced angiogenesis both in vitro and in vivo [113]. A recent study identified a direct interaction between PMCA4 and the transmembrane glycoprotein CD147, a molecule frequently overexpressed in human cancers and implicated in tumor invasion and metastasis. Although this work was performed in activated T lymphocytes rather than cancer cells, it demonstrated that PMCA4 cooperates with CD147 to regulate calcium-dependent signaling downstream of receptor activation, thereby uncoupling early signaling events from IL-2 production. Given the well-established role of CD147 in tumor biology, these findings suggest that PMCA4 may also contribute to cancer progression through regulation of membrane signaling platforms independently of its calcium transport activity [192].

Overall, the seemingly contradictory roles of PMCA4 in cancer can be reconciled by considering its function as a context-dependent organizer of localized Ca^2+^ signaling rather than simply a Ca^2+^ extrusion pump. PMCA4 does not intrinsically promote or suppress tumor progression. Instead, its biological effect depends on the signaling environment in which it operates. Factors including tumor lineage, subcellular localization within specialized membrane microdomains, splice-variant expression, availability of interacting proteins, and the architecture of PMCA4-containing signaling complexes collectively determine which Ca^2+^-dependent pathways are preferentially regulated. This framework explains why PMCA4 can suppress proliferation and metastasis in some malignancies while facilitating migration, invasion, or therapy resistance in others, and highlights the importance of developing context-specific strategies for targeting PMCA4 in precision oncology.

## 9. Current Strategies for Therapeutic Targeting of PMCAs

The growing body of evidence linking PMCA isoforms to tumor initiation, progression, metastasis, and therapy resistance has stimulated considerable interest in exploiting these calcium pumps as therapeutic targets. However, despite compelling experimental data, no PMCA-directed therapy has yet advanced to clinical evaluation. This reflects the fundamental physiological importance of PMCAs, their widespread tissue distribution, and the difficulty of selectively targeting individual isoforms without disrupting systemic calcium homeostasis. Consequently, current therapeutic approaches have largely focused on developing experimental tools that either inhibit PMCA activity directly or interfere with isoform-specific signaling complexes.

The best-characterized pharmacological inhibitors of PMCAs are the caloxins, a family of extracellular peptide inhibitors identified through phage-display screening [193]. Caloxins bind to extracellular loops of the PMCA molecule and inhibit calcium extrusion without entering the cell. Successive generations of caloxins have demonstrated increasing affinity toward individual PMCA isoforms, providing valuable experimental tools for dissecting PMCA biology. Nevertheless, their therapeutic potential remains limited because of relatively low potency, incomplete isoform selectivity, poor pharmacokinetic properties, and the absence of in vivo efficacy studies. At present, caloxins should therefore be regarded primarily as research tools rather than clinically applicable anticancer agents.

An emerging concept is that selective disruption of PMCA-associated signaling complexes may provide greater therapeutic specificity than inhibition of calcium transport itself. This strategy is exemplified by the competitive peptide developed by Baggott and colleagues [154], which disrupts the interaction between PMCA2 and calcineurin without affecting pump activity. By releasing calcineurin from the inhibitory PMCA2 complex, the peptide restored calcineurin/NFAT signaling and demonstrated that PMCA scaffolding functions can be selectively manipulated independently of global calcium extrusion. Although this approach has so far been validated only in experimental systems, it provides an important proof of principle that targeting PMCA protein–protein interactions may represent a viable alternative to direct pump inhibition.

Another promising strategy exploits the dependence of oncogenic signaling pathways on PMCA-containing membrane complexes. In HER2-positive breast cancer, PMCA2 forms a functional complex with HER2 and the cytoskeletal adaptor ezrin, thereby stabilizing HER2 signaling at the plasma membrane [148]. Pharmacological inhibition of ezrin disrupted this complex and markedly enhanced the antitumor activity of the HER2 inhibitor lapatinib, providing one of the strongest translational examples of PMCA-targeted combination therapy. Rather than directly inhibiting PMCA2 catalytic activity, this approach selectively destabilizes oncogenic signaling platforms that depend on PMCA-mediated spatial organization, thereby potentially reducing systemic toxicity.

Recent structural and biochemical studies have also identified plasma membrane lipids as potential modulators of PMCA function. High-resolution cryo-electron microscopy demonstrated that PIP_2_ directly interacts with PMCA2 and stabilizes functionally important conformational states of the transporter [18]. Similarly, the localization of PMCA4 within cholesterol-rich lipid rafts has been shown to be essential for the formation of signaling complexes regulating localized calcium microdomains in glioma cells [190]. These observations suggest that modulation of membrane lipid composition or disruption of lipid raft integrity could indirectly alter PMCA activity and selectively interfere with compartmentalized calcium signaling in cancer cells. Although such approaches remain at an early experimental stage, they may ultimately provide greater specificity than global inhibition of PMCA-mediated calcium transport.

Collectively, current therapeutic strategies targeting PMCAs remain at the preclinical stage and can be broadly classified into four categories: direct inhibition of pump activity, disruption of PMCA-dependent protein–protein interactions, destabilization of oncogenic signaling complexes, and modulation of membrane lipid environments that regulate PMCA function. Future advances in structural biology, medicinal chemistry, peptide engineering, and targeted protein degradation technologies may facilitate the development of isoform-selective therapeutics capable of exploiting PMCA-dependent signaling pathways while minimizing adverse effects associated with inhibition of this essential calcium transport system.

## 10. Conclusions and Future Perspectives

Our understanding of PMCAs has evolved substantially. Once regarded primarily as housekeeping proteins responsible for maintaining low intracellular Ca^2+^ concentrations, PMCAs are now recognized as dynamic regulators of compartmentalized calcium signaling that integrate ion transport with the control of multiple signaling pathways. This functional complexity is particularly evident in cancer, where alterations in PMCA expression, activity, alternative splicing, and protein–protein interactions contribute to the extensive remodeling of calcium signaling that accompanies malignant transformation. As highlighted throughout this review, PMCA isoforms influence numerous hallmarks of cancer, including proliferation, apoptosis, migration, invasion, epithelial plasticity, angiogenesis, immune regulation, and therapeutic resistance. However, their biological roles are highly context dependent, varying according to isoform, splice variant, subcellular localization, tumor type, and the surrounding signaling environment. Consequently, PMCA proteins cannot be simply classified as oncogenes or tumor suppressors.

Collectively, the available evidence reveals a common mechanistic principle underlying the diverse roles of PMCA isoforms in cancer. PMCA1 primarily maintains basal Ca^2+^ homeostasis while contributing to angiogenesis and tumor–microenvironment interactions. PMCA2 promotes oncogenic signaling in selected malignancies, particularly HER2-positive breast cancer, through stabilization of receptor signaling complexes and localized Ca^2+^ regulation. In contrast, current evidence for PMCA3 remains limited and is derived mainly from endocrine tumors, highlighting the need for further mechanistic studies. PMCA4 exhibits the greatest functional diversity, functioning either as a tumor suppressor or a tumor promoter depending on the molecular context. Importantly, these apparently divergent roles are best explained not by differences in global Ca^2+^ extrusion but by the ability of PMCA isoforms to generate distinct compartmentalized Ca^2+^ signaling microdomains and assemble isoform-specific signaling complexes. Thus, the biological consequences of PMCA activity are determined by the signaling environment in which individual isoforms operate rather than by calcium transport alone.

This emerging paradigm also has important therapeutic implications. Because PMCA isoforms are widely expressed and play indispensable physiological roles in numerous tissues, the clinical success of PMCA-targeted therapies will likely depend on achieving high isoform specificity or selectively disrupting disease-specific PMCA signaling complexes while minimizing systemic toxicity. Rather than indiscriminately inhibiting PMCA activity, future therapeutic strategies should therefore focus on selectively targeting the signaling functions of individual PMCA isoforms. Promising approaches include disrupting PMCA-dependent protein–protein interactions, modulating alternative splice variants, interfering with PMCA localization within specialized membrane microdomains, and targeting signaling complexes that couple PMCAs to oncogenic pathways such as HER2, calcineurin/NFAT, or nitric oxide signaling. By preserving the essential physiological functions of PMCAs in normal tissues while selectively disrupting their pathological activities in cancer cells, these strategies may offer improved therapeutic specificity and a wider therapeutic window.

In summary, PMCAs have emerged as multifunctional regulators of cancer biology whose activities extend far beyond calcium extrusion. Their ability to shape localized calcium signaling, assemble signaling complexes, and coordinate multiple oncogenic pathways places them at the center of the increasingly recognized relationship between calcium homeostasis and tumor progression. Continued investigation of PMCA biology will not only advance our understanding of calcium signaling in cancer but may also identify novel biomarkers and therapeutic opportunities for precision oncology.

## Figures and Tables

**Table 1:** Alternative splicing at sites A and C generates numerous PMCA variants that differ in their regulatory properties, intracellular targeting, and physiological functions. The distinct expression patterns and knockout phenotypes of individual PMCA isoforms demonstrate their nonredundant roles in calcium homeostasis, neuronal signaling, sensory function, cardiovascular physiology, and reproduction [19,20,21,22].

Isoform/Gene	Major Splice Variants	Expression and Tissue Distribution	Subcellular Localization	Mouse Knockout Phenotype	Disease/Clinical Relevance
PMCA1 (ATP2B1)	x/a, x/b, x/c, x/d	Ubiquitously expressed; particularly abundant in brain, vascular smooth muscle, heart, and skeletal muscle	Plasma membrane; predominantly basolateral and lateral membrane domains in polarized cells	Homozygous deletion causes embryonic lethality; heterozygous animals exhibit altered vascular smooth muscle Ca^2+^ handling and blood pressure regulation	ATP2B1 polymorphisms are strongly associated with hypertension and cardiovascular disease risk
PMCA2 (ATP2B2)	w/a, x/a, z/a, w/b, x/b, z/b	Highly enriched in excitable tissues; cerebellum, cochlear and vestibular hair cells, retina, mammary gland	Apical membrane of polarized epithelia; synaptic membranes; sensory-cell microdomains	Profound deafness, severe ataxia, impaired balance, reduced milk calcium content, motor neuron degeneration, altered cerebellar plasticity	Mutations in ATP2B2 are associated with hereditary hearing loss, vestibular dysfunction, and neurological disorders
PMCA3 (ATP2B3)	x/a, z/a, x/b, z/b, x/f, z/f	Predominantly expressed in neurons; also found in pancreatic β-cells and fast skeletal muscle	Neuronal plasma membrane; specialized calcium signaling microdomains	No comprehensive knockout phenotype reported; likely compensated by other PMCA isoforms	ATP2B3 mutations cause X-linked cerebellar ataxia, developmental delay, hypotonia, and motor dysfunction
PMCA4 (ATP2B4)	x/a, z/a, x/b, z/b	Widely expressed; abundant in heart, brain, stomach, testis, immune cells, and vascular tissues	Plasma membrane; lipid rafts, caveolae, sperm flagellum, signaling domains	Male infertility due to impaired sperm motility; altered vascular smooth muscle Ca^2+^ signaling, particularly in combination with PMCA1 deficiency	Associated with male infertility, cardiovascular regulation, immune signaling, and potentially cancer progression

**Table 2:** Selected Kinetic Properties of PMCA Isoforms and Splice Variants [20,28,30,31,32]. The kinetic parameters summarized in this table were compiled from independent studies employing different experimental models, expression systems, and assay conditions. Consequently, the values should be interpreted as representative characteristics of individual PMCA isoforms and splice variants rather than as directly comparable quantitative measurements.

PMCA	Activation Kinetics	Deactivation Kinetics	Basal Activity	Functional Specialization	Typical Expression
PMCA1b	Moderate	Moderate	Low–moderate	Maintenance of basal intracellular Ca^2+^ levels; housekeeping function	Most tissues
PMCA2b	Very fast	Slow	High	Rapid clearance of repetitive Ca^2+^ transients; efficient decoding of high-frequency Ca^2+^ spikes	Neurons, cochlear hair cells, retina
PMCA2a	Extremely fast	Relatively fast	Very high	Immediate response to brief Ca^2+^ elevations; sensory signaling	Hair cells, sensory neurons
PMCA3f	Very fast	Intermediate	High	Regulation of rapid neuronal and muscle Ca^2+^ signals	Brain, fast skeletal muscle
PMCA3b	Fast	Slow–intermediate	Moderate	Sustained regulation of neuronal Ca^2+^ signaling	Neurons
PMCA4a	Fast (t½ ≈ 20 s for CaM activation)	Intermediate	High	Efficient attenuation of agonist-induced Ca^2+^ transients	Heart, brain, smooth muscle
PMCA4b	Slow (t½ ≈ 1 min for CaM activation)	Very slow (t½ ≈ 20 min)	Lower than PMCA4a	Predicted to support sustained regulation of prolonged or tonic Ca^2+^ signals based on its kinetic properties	Ubiquitous
PMCA4x/b	Slow–moderate	Very slow	Moderate	Formation of signaling complexes and regulation of localized Ca^2+^ microdomains	Many cell types

**Table 3:** PMCA-Interacting Proteins and Their Functional Significance in Signal Transduction. Based on [94]. nNOS (NOS-1)—Neuronal Nitric Oxide Synthase; CASK—Calcium/Calmodulin-Dependent Serine Protein Kinase; CLP36—CLP36 Actin-Associated LIM Domain Protein; MAGUK—Membrane-Associated Guanylate Kinase family proteins; NHERF-2—Na^+^/H^+^ Exchanger Regulatory Factor 2; Ania-3—Activity-Induced Immediate Early Gene Product 3; Homer—Homer Scaffolding Protein Family; RASSF1—Ras Association Domain Family Member 1; α1-Syntrophin—Cytoskeletal Adaptor Protein of the Dystrophin Complex; 14-3-3ε—Epsilon Isoform of the 14-3-3 Regulatory Protein Family.

Interacting Protein	PMCA Domain Involved in Interaction	Partner Protein Domain Involved in Interaction	Functional Consequence of the Interaction
nNOS (NOS-1)	PDZ-binding motif	PDZ domain	Inhibition of nNOS activity and reduction in nitric oxide (NO) production
CASK	PDZ-binding motif	PDZ domain	Decreased T-element-dependent transcriptional activity
CLP36	PDZ-binding motif	PDZ domain	PMCA translocation during platelet activation
MAGUK family proteins	PDZ-binding motif	PDZ domain	Targeting of PMCA to specific membrane domains and local regulation of Ca^2+^ concentrations
NHERF-2	PDZ-binding motif	PDZ domain	Stabilization of PMCA within specialized cellular microdomains
Ania-3/Homer	PDZ-binding motif	PDZ domain	Retention of PMCA near sites of Ca^2+^ influx, facilitating local calcium control
eNOS (NOS-3)	Catalytic domain	Amino acids 735–935	Suppression of eNOS activity and decreased NO synthesis
Calcineurin	Catalytic domain	Amino acids 58–143	Inhibition of calcineurin phosphatase activity and reduced NFAT-dependent transcription
RASSF1	Catalytic domain	Amino acids 74–123 or 144–193	Attenuation of EGF-induced ERK signaling pathway activation
α1-Syntrophin	Catalytic domain	Amino acids 399–447	Formation of a PMCA–α1-syntrophin–nNOS complex that suppresses NO production
14-3-3ε	N-terminal region	Amino acids 2–92	Inhibition of PMCA activity

**Table 4:** Evidence supporting PMCA1 protein function versus cancer-associated non-coding transcripts derived from the ATP2B1 locus. Studies investigating PMCA1 protein expression or ATP2B1 mRNA provide direct evidence for the involvement of PMCA1 in tumor biology, whereas investigations of ATP2B1-AS1 and circATP2B1 describe independent regulatory non-coding RNAs transcribed from the same genomic locus.

Biological Entity	Cancer Type/Model	Principal Findings	Implications
PMCA1 protein/ATP2B1 expression	Oral squamous cell carcinoma	Reduced ATP2B1 expression compared with normal epithelium	Supports a direct role of PMCA1 in maintaining Ca^2+^ homeostasis during malignant transformation
PMCA1 protein/ATP2B1 expression	Endothelial cells	ATP2B1 silencing impairs cell survival, migration, and angiogenesis	Suggests PMCA1 contributes to tumor vascularization and progression
PMCA1 protein/ATP2B1 expression	Intrahepatic cholangiocarcinoma	High ATP2B1 expression correlates with immune infiltration, low promoter methylation, and favorable prognosis	Supports PMCA1 as a prognostic biomarker associated with an immune-active tumor microenvironment
PMCA1 protein/ATP2B1 expression	Cholangiocarcinoma (multi-omics)	ATP2B1 identifies molecular subtype with increased responsiveness to immune checkpoint blockade	Supports clinical relevance of ATP2B1 expression
PMCA1 protein/ATP2B1 expression	Breast cancer	ATP2B1 identified among genes associated with disease progression	Suggests involvement of PMCA1-associated signaling networks, although direct functional evidence is limited
PMCA1 protein/ATP2B1 expression	Laryngeal squamous cell carcinoma	ATP2B1 predicted as a target of a three-miRNA regulatory network	Bioinformatic evidence only; no direct evidence for PMCA1 protein function
ATP2B1-AS1 (lncRNA)	Gastric cancer	Prognostic lncRNA regulating the miR-425-3p/ZC3H12A axis and immune evasion	Represents ATP2B1 locus biology rather than PMCA1 protein function
circATP2B1 (circRNA)	Gastric cancer	Promotes aerobic glycolysis through the miR-326 pathway	Independent function of a circular RNA derived from the ATP2B1 locus
ATP2B1-AS1 (lncRNA)	Lung adenocarcinoma	Overexpression associated with tumor progression	ncRNA-mediated regulation; not evidence for PMCA1 pump activity
ATP2B1-AS1 (lncRNA)	Esophageal squamous cell carcinoma	Component of an m^5^C-related prognostic signature associated with immune landscape and survival	Biomarker derived from the ATP2B1 locus
ATP2B1-AS1 (lncRNA)	Colorectal cancer	Increased expression associated with poor prognosis; validated by RT-qPCR and WGCNA	Prognostic lncRNA independent of demonstrated PMCA1 protein function

**Table 5:** Context-dependent mechanisms of PMCA2 in cancer.

Cancer Type/Model	Principal PMCA2 Function	Molecular Mechanism	Biological Outcome
HER2-positive breast cancer	Stabilization of HER2 signaling	PMCA2–HER2–ezrin complex maintains HER2 phosphorylation	Increased proliferation, survival, poor prognosis
MDA-MB-231 breast cancer	Maintenance of proliferation	Regulation of intracellular Ca^2+^ during cell-cycle progression	Reduced proliferation after PMCA2 silencing
MDA-MB-231 breast cancer	Anti-apoptotic function	Limitation of Ca^2+^ overload during stress	Increased apoptotic sensitivity after PMCA2 knockdown
Lactating mammary gland/breast cancer	Calcineurin regulation	PMCA2 binds and inhibits calcineurin	Regulation of NFAT signaling
HER2-positive breast cancer	Therapeutic target	Ezrin inhibition destabilizes PMCA2/HER2 complex and enhances lapatinib response	Increased drug sensitivity

## Data Availability

No new data were created or analyzed in this study.

## Abbreviations

The following abbreviations are used in this manuscript:

PMCA	Plasma Membrane Ca^2+^-ATPase
HER2	Human Epidermal Growth Factor Receptor 2
SERCA	Sarco/endoplasmic reticulum Ca^2+^-ATPase
NCX	Na^+^/Ca^2+^ exchanger
PIP_2_	Phosphatidylinositol 4,5-bisphosphate
CaM	Calmodulin
PKA	Protein kinase A
PKC	Protein kinase C
nNOS	Neuronal nitric oxide synthase
NFAT	Nuclear factor of activated T cells
CASK	Calcium/Calmodulin-Dependent Serine Protein Kinase
PSD-95	Postsynaptic density protein 95
RASSF	Ras Association Domain Family Member 1
MAGUK	Membrane-Associated Guanylate Kinase family proteins
ZC3H12A	Zinc Finger CCCH-Type Containing 12A
CaSR	Calcium-sensing receptor
PTHrP	Parathyroid hormone-related protein
EGFR	Epidermal Growth Factor Receptor
AKT	Protein kinase B
HSP90	Heat Shock Protein 90
NHERF1	Na^+^/H^+^ Exchanger Regulatory Factor 1
SQLE	Squalene epoxidase
APA	Aldosterone-producing adrenal adenoma
KCNJ5	Potassium Inwardly Rectifying Channel Subfamily J Member 5
ATP1A1	ATPase Na^+^/K^+^ Transporting Subunit Alpha 1
CACNA1D	Calcium Voltage-Gated Channel Subunit Alpha1 D
CYP11B2	Aldosterone synthase
CTNNB1	β-catenin
ACTH receptor	Adrenocorticotropic hormone receptor
FOS	Fos Proto-Oncogene, AP-1 Transcription Factor Subunit
TRAIL	Tumor Necrosis Factor-Related Apoptosis-Inducing Ligand
MAPK	Mitogen-Activated Protein Kinase
MEK	Mitogen-Activated Protein Kinase
BRAF	B-Raf Proto-Oncogene, Serine/Threonine Kinase
HDAC	Histone Deacetylase
PRKCA	Protein Kinase C Alpha
PDAC	Pancreatic ductal adenocarcinoma
EMT	Epithelial–Mesenchymal Transition
ZEB1	Zinc Finger E-Box Binding Homeobox 1
GAT3	Gamma-Aminobutyric Acid Transporter 3
CAMKII	Calcium/Calmodulin-Dependent Protein Kinase II
ACYP2	Acylphosphatase 2
STAT3	Signal Transducer and Activator of Transcription 3
VEGF	Vascular Endothelial Growth Factor
RCAN1.4	Regulator of Calcineurin 1 isoform 4
COX-2	Cyclooxygenase-2
CD147	Cluster of Differentiation 147
IL-2	Interleukin-2
